# Physicochemical Characterization, and Relaxometry Studies of Micro-Graphite Oxide, Graphene Nanoplatelets, and Nanoribbons

**DOI:** 10.1371/journal.pone.0038185

**Published:** 2012-06-07

**Authors:** Bhavna S. Paratala, Barry D. Jacobson, Shruti Kanakia, Leonard Deepak Francis, Balaji Sitharaman

**Affiliations:** 1 Department of Biomedical Engineering, Stony Brook University, Stony Brook, New York, United States of America; 2 International Iberian Nanotechnology Laboratory, Avda Mestre Jose Veiga, Braga, Portugal; National Institute of Health, United States of America

## Abstract

The chemistry of high-performance magnetic resonance imaging contrast agents remains an active area of research. In this work, we demonstrate that the potassium permanganate-based oxidative chemical procedures used to synthesize graphite oxide or graphene nanoparticles leads to the confinement (intercalation) of trace amounts of Mn^2+^ ions between the graphene sheets, and that these manganese intercalated graphitic and graphene structures show disparate structural, chemical and magnetic properties, and high relaxivity (up to 2 order) and distinctly different nuclear magnetic resonance dispersion profiles compared to paramagnetic chelate compounds. The results taken together with other published reports on confinement of paramagnetic metal ions within single-walled carbon nanotubes (a rolled up graphene sheet) show that confinement (encapsulation or intercalation) of paramagnetic metal ions within graphene sheets, and not the size, shape or architecture of the graphitic carbon particles is the key determinant for increasing relaxivity, and thus, identifies nano confinement of paramagnetic ions as novel general strategy to develop paramagnetic metal-ion graphitic-carbon complexes as high relaxivity MRI contrast agents.

## Introduction

Magnetic chemical compounds called contrast agents (CA) are widely used, and nowadays integral to improve the detection and diagnostic confidence of Magnetic resonance imaging (MRI); one of the central non-invasive imaging modalities in radiology used to provide anatomical details of various organs and tissues for improved diagnosis of pathologies and diseases. The chemistry and design of contrast agents remains an active area of research in academia and industry [1]–[3]. The two main types are *T*_1_ and *T*_2_ MRI CAs, and affect (decrease) the longitudinal *T*_1_ and transverse *T*_2_ relaxation times of water protons, respectively. The quantitative measure of their effectiveness to accelerate the relaxation process of the water protons is known as relaxivity; the change in relaxation rate (inverse of relaxation time) per unit concentration of the MRI CA. The widely-used clinical *T*_1_ MRI CAs are mainly synthesized as metal-ion chelate complexes, where the metal ion is the lanthanoid element gadolinium (Gd^3+^), or the inner-transition element manganese (Mn^2+^). A large body of experimental and theoretical research done in the last three decades now offers good understanding of the relaxation mechanism, and underlying structural, chemical and molecular dynamic properties that influence the relaxivity of these paramagnetic-ion chelate complexes [1]–[3]. Theory suggests that the relaxivity of these MRI contrast agents is sub-optimal, and predicts the possibility of developing new contrast agents up to at least fifty to hundred times greater relaxivity [4], [5].

Over the past decade, Gd^3+^- ion carbon nanostructure complexes have been developed as MRI CAs [6]. The synthesis strategies in the development of these complexes have focused on covalently or non-covalently functionalizing multiple Gd^3+^-chelate complexes onto the external carbon sheet of carbon nanostructures such as carbon nanotubes and nanodiamonds [7], [8], or encapsulation of Gd^3+^- ions within the carbon sheet of carbon nanostructures such as fullerene (a.k.a. gadofullerenes) [9]–[11], and single-walled carbon nanotubes (a.k.a. gadonanotubes) [12], [13]. These Gd^3+^- ion carbon nanostructures show between two-fold to two-order increase in relaxivity (depending on the magnetic field) compared to Gd^3+^-chelate complexes with the gadonanotubes showing the highest relaxivities at low to high (0.01-3T) magnetic fields. However, the potential and efficacy of Mn^2+^- ion carbon nanostructure complexes as MRI CAs still has not been investigated.

The variable-magnetic field (0.01-3T) relaxivity or nuclear magnetic resonance dispersion (NMRD) profiles of the gadonanotubes are characteristically different than those obtained for any other MRI CA and their relaxation mechanisms are not well understood. A major reason for this lack of understanding is that unlike Gd^3+^ ion chelates, which can be prepared at a very high level of purity and unambiguously characterized, the carbon nanostructure-Gd^3+^ ion systems are rather complex mainly due to their particulate nature, and intricate relationships linking their chemical, geometric, and magnetic characteristics to their properties as MRI contrast agents. Nevertheless, geometric confinement of the Gd^3+^ ion within nanoporous structures may be one reason [13], [14]. While confinement of the Gd^3+^ ions into nanoporous structures of silicon [13] or zeolites [14] increases the relaxivity by two or four times compared to Gd^3+^ small molecule chelate compounds (e.g. gadolinium-diethylenetriaminepentaacetic acid or Gd-DTPA), only when the Gd^3+^ ions are confined within single-walled carbon nanotubes [12], [13] has there been an order of magnitude or more increase in relaxivity (irrespective of the magnetic field strength) with NMRD profiles significantly different that those reported for other Gd^3+^ ion-based complexes. Additionally, to date, there have been no studies performed to systematically investigate whether the high increase in relaxivity and unconventional NMRD profiles are unique to paramagnetic ions confined in single-walled carbon nanotubes, which are seamless cylinders formed from a graphene *s*heet, or in general observed for paramagnetic ions confined in other graphene or graphitic structures.

Graphene, a two-dimensional (2-D) nanostructure of carbon, has attracted a great deal of attention, and has shown potential for various material and biomedical science applications [15]. Theoretical studies predict a variety of magnetic phenomena in graphene [16], and to date, few of these effects have been explored experimentally [17]. Recently, simple potassium permanganate (KMnO_4_)-based oxidative chemical procedures have been used in the large scale production of graphite oxide, graphene nanoplatelets, and graphene nanoribbons using starting materials such as graphite and MWCNTs [18], [19]. In this work, experimental studies were performed to characterize the physico-chemical properties of graphite oxide, graphene nanoplatelets, and graphene nanoribbons synthesized using these techniques. We demonstrate that trace amounts of Mn^2+^ ions become confined (intercalated) within the graphene sheets during the synthesis process, and that this confinement in general substantially increases the relaxivity (up to 2 order) compared to paramagnetic chelate compounds, and these materials show diverse structural, chemical and magnetic properties with NMRD profiles different than those of the paramagnetic chelates.

## Materials and Methods

### 1. Graphene Nanoplatelets and Nanoribbons Synthesis

A total of 5 batches of graphene nanoplalets and nanoribbons were prepared and characterized. All the results presented except the relaxivity results are representative data of a single batch. Oxidized micro-graphite was prepared from analytical grade micro-graphite (Sigma Aldrich, New York) by modified Hummer’s method [20], [21]. In a typical exfoliation procedure, dried oxidized micro-graphite (200 mg) was suspended in a round bottom flask containing water (200 ml) and sonicated for 1 h in an ultrasonic bath cleaner (Fischer Scientific, FS60, 230 W). 50 ml of this uniform solution was centrifuged and pellet was dried overnight to obtain oxidized graphene nanoplatelets. The remaining 150 ml was treated with hydrazine hydrate (1.5 ml, 37.1 mmol), and heated in an oil bath at 100°C under a water cooled condenser for 12 h, resulting in a black precipitate. The product was isolated, and washed over a medium sintered glass filter funnel with water (500 ml) and methanol (500 ml) and dried by continuous air flow to yield reduced graphene nanoplatelets.

**Figure 1:**
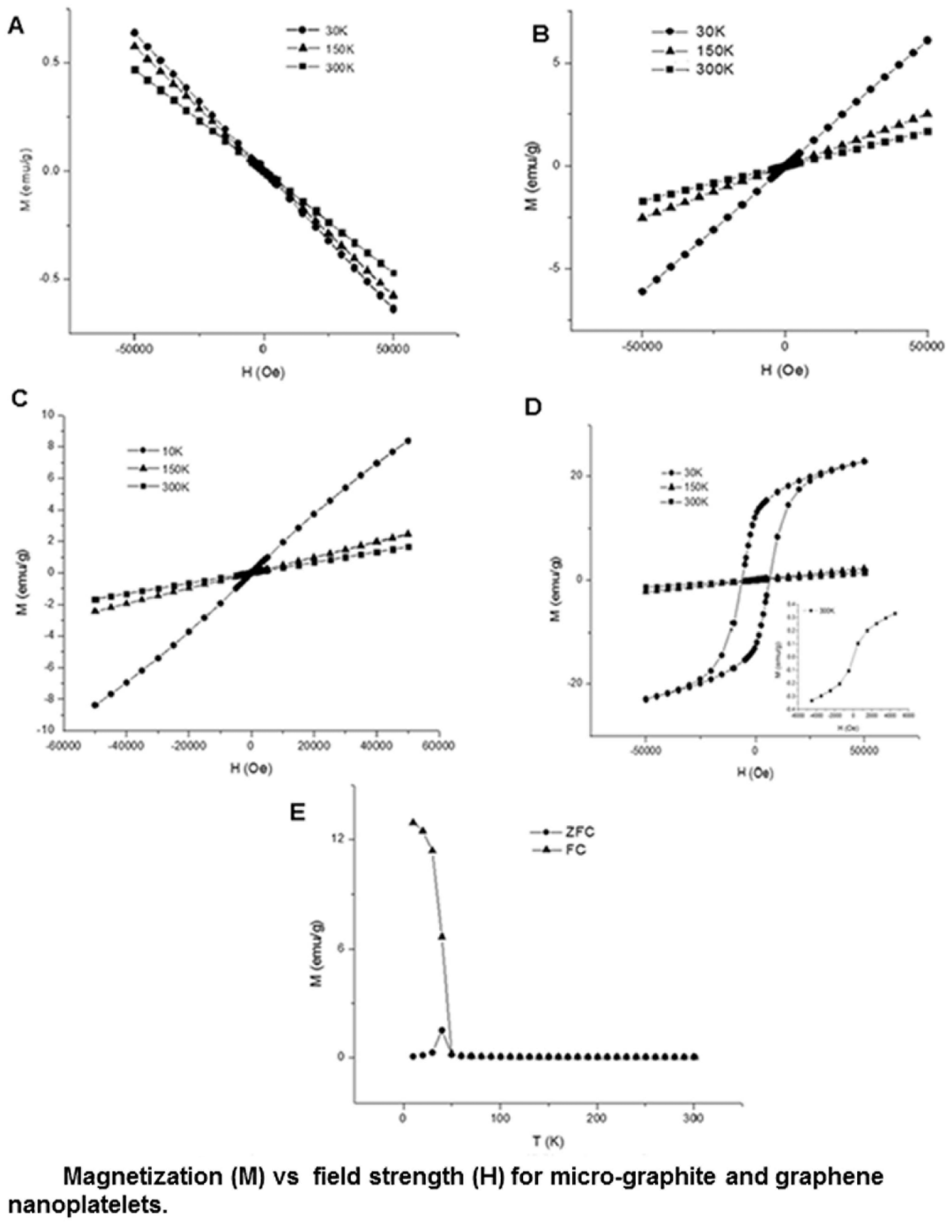
Plot of Magnetization (M) v/s Field strength (H) for (a) micro-graphite, (b) oxidized graphite (c) Oxidized Graphene nanoplatelets (d) Reduced Graphene nanoplatelets at 30 K, 150 and 300 K between −50,000 to 50,000 Oe (Inset shows plot between −5000 and 5000 Oe at 300 K), (e) ZFC and FC magnetization plots of reduced graphene nanoplatelets.

Graphene nanoribbons were prepared from MWCNTs (Sigma Aldrich, New York) in a procedure similar to the one previously described [19], [22]. MWCNTs (150 mg, 12.5 mequiv of carbon) were suspended in 30 ml of conc. H_2_SO_4_ for 2 h. KMnO_4_ (750 mg, 4.75 mmol) was added, and the mixture was allowed to stir for 1 h. The reaction was then heated in an oil bath at 55–70°C for an additional 1 h, until completion. It was cooled to room temperature, and the product was washed with water, ethanol and ether, and subsequently isolated by centrifugation.

**Figure 2:**
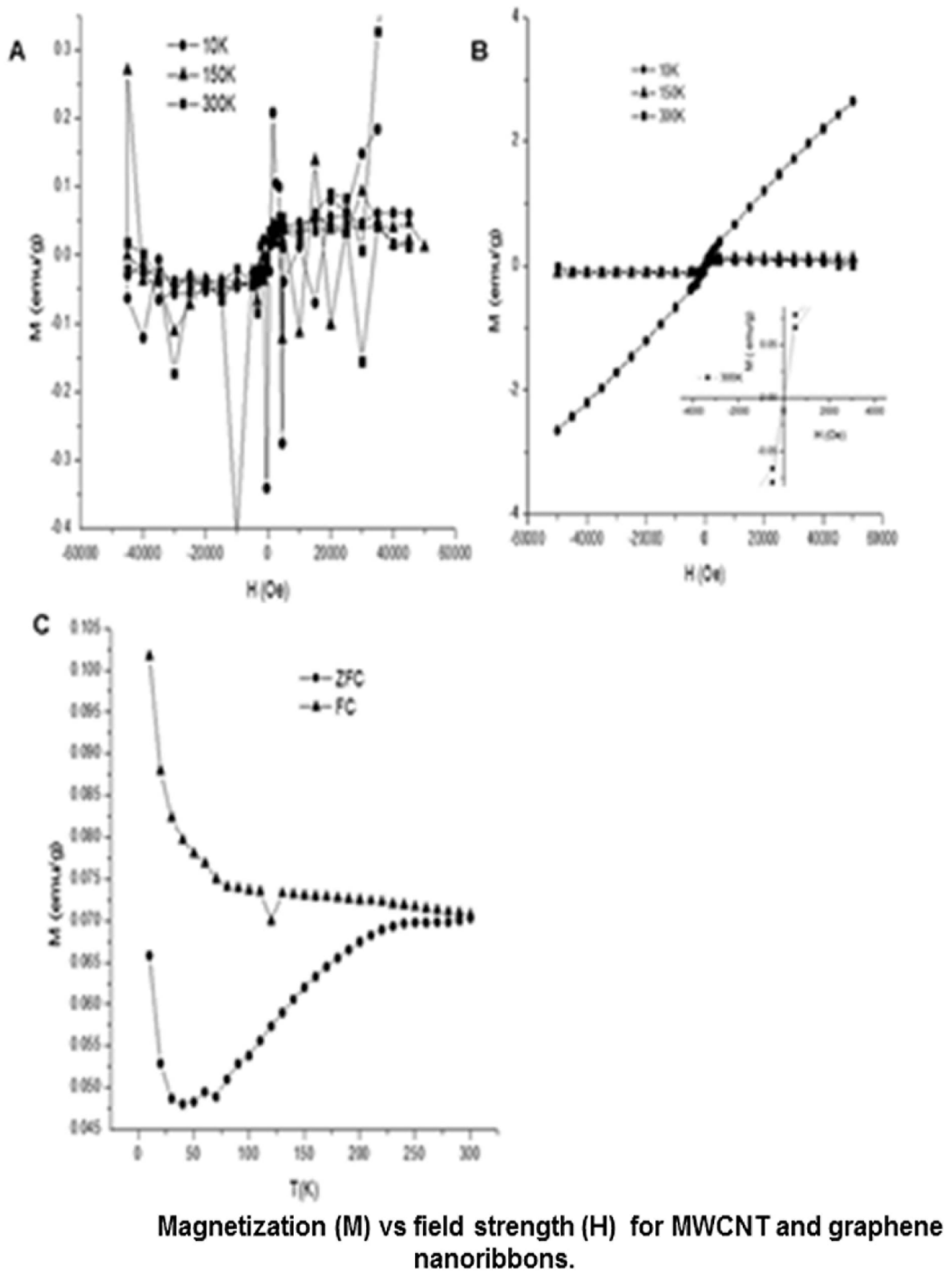
Magnetization (M) v/s Field strength (H) between −50,000 Oe and 50,000 Oe at 10, 150 and 300 K for (a) MWCNTs, and (b) graphene nanoribbons (Inset shows M versus H between −4000 Oe and 4000 Oe at 300 K), (c) ZFC and FC plots of graphene nanoribbons.

The solid and liquid graphene nanoplatelets and nanoribbon samples were analyzed by Inductively-coupled plasma optical emission spectroscopy (ICP-OES) (see table S1 and S2 for details) to confirm, and determine the concentration of manganese and potassium. Additionally, iron content analysis was carried out for the graphene nanoribbon samples, since iron is used as a catalyst in the preparation of MWCNTs (the starting material).

**Figure 3:**
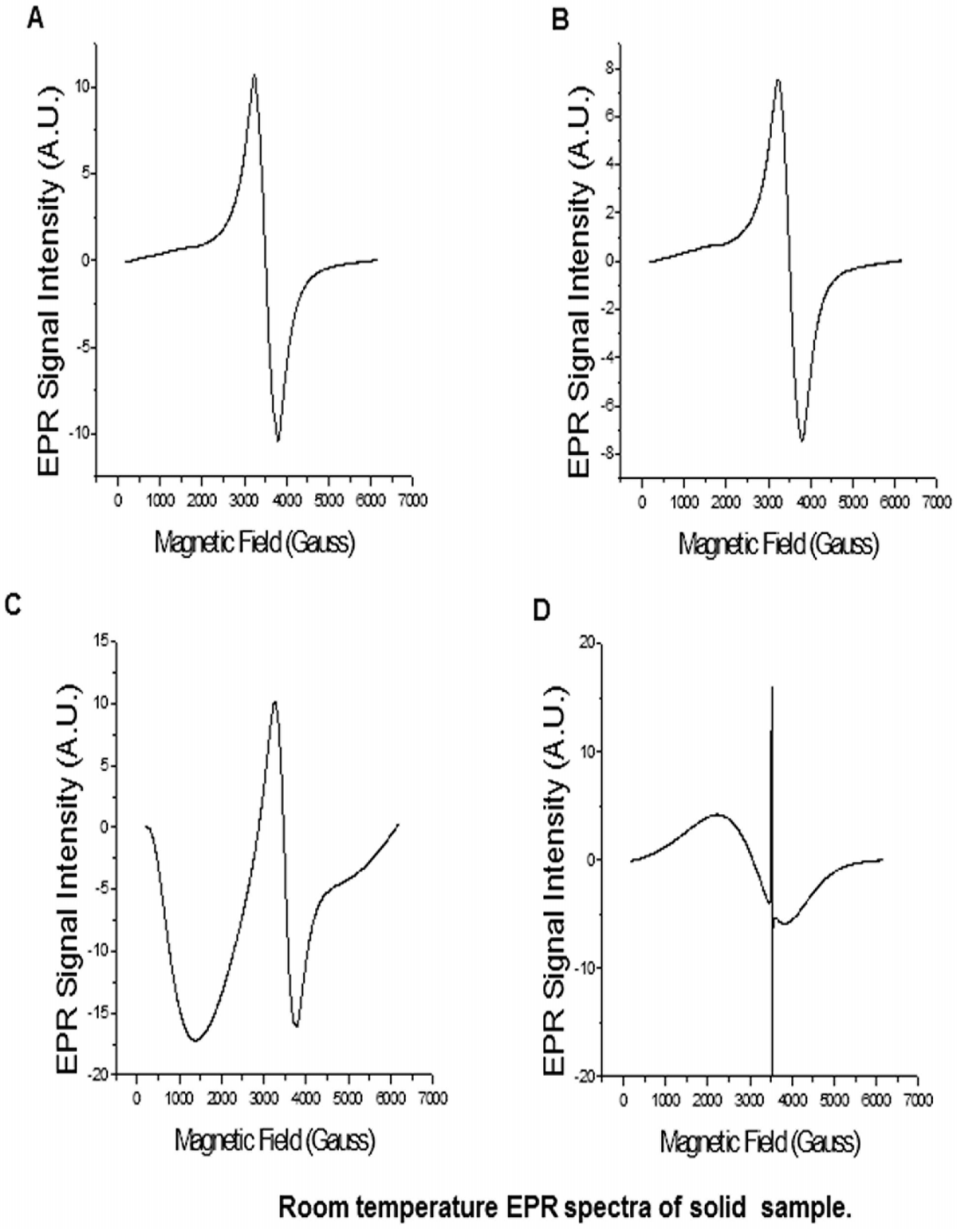
Room temperature EPR spectra of solid (a) oxidized micro-graphite, (b) oxidized graphene nanoplatelets, (c) reduced graphene nanoplatelets and (d) graphene nanoribbons.

**Table 1:** EPR parameters of solid samples of oxidize micro-graphite, oxidized graphene nanoplatelets, reduced graphene nanoplatelets and graphene nanoribbons.

Sample	g-value	EPR Line width (ΔH_1/2_, Gauss)for g∼2.0	Electron relaxation time(*T_2e_*, nanoseconds)
Oxidized micrographite	2.007	552.0	0.19
Oxidized graphene nanoplatelets	2.007	544.4	0.20
Reduced graphene nanoplatelets	2.008	505.2	0.21
Graphene nanoribbons	2.313	1472.0	88.2

### 2. Characterization of Magnetic Behavior

Magnetization of graphite, graphene and control samples was studied using a super conducting quantum interference device (SQUID) magnetometer with a sensitivity of about 10^−8 ^emu. The samples were carefully weighed and loaded in gelatin capsules. Samples were analyzed between the applied magnetic field range of −50000 Oe to 50000 Oe between 0 and 300 K. In the Field cooling and Zero Field cooling mode, a coercive field of 500 Oe was applied for studying magnetization as a function of temperature.

**Figure 4:**
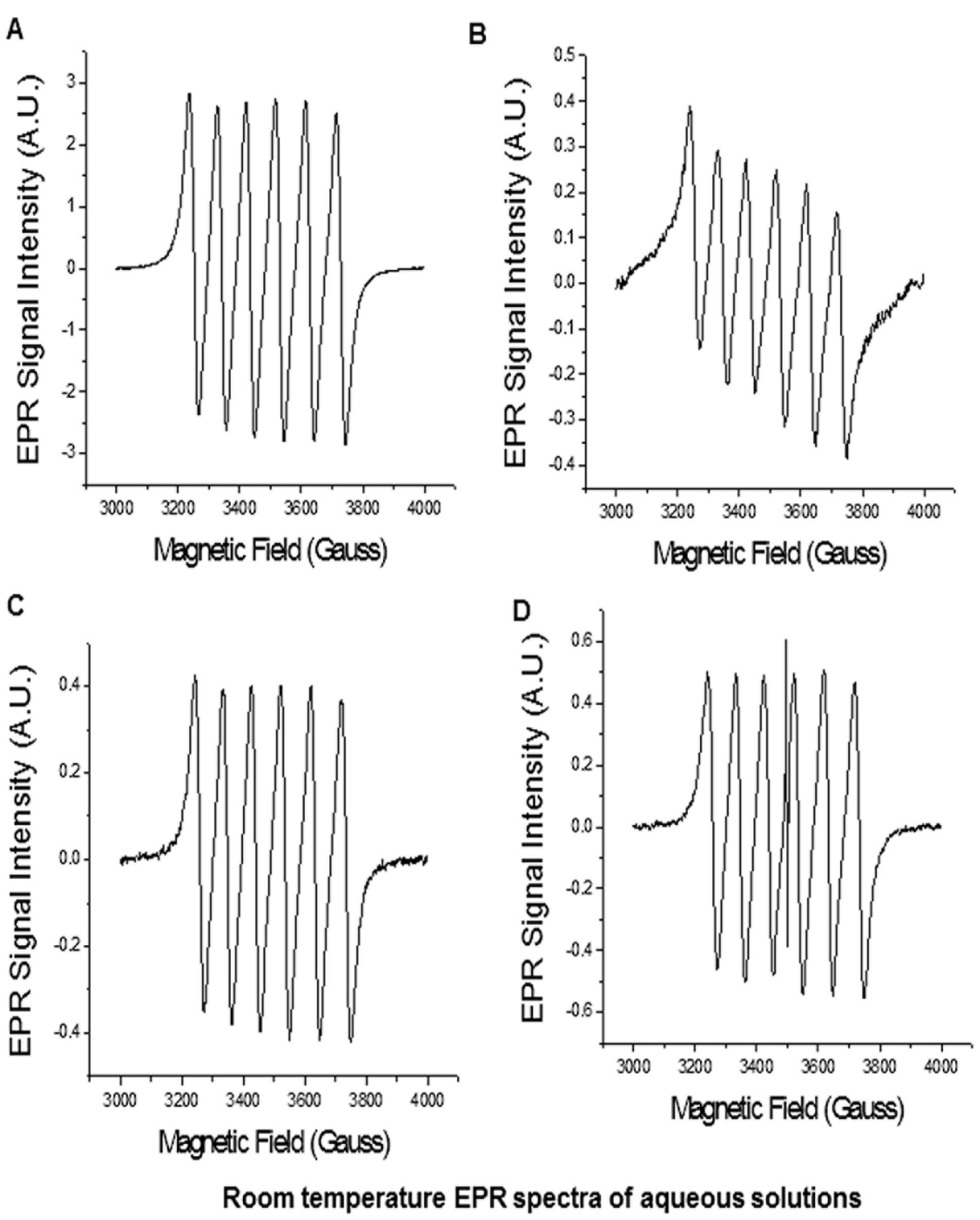
Room temperature EPR spectra of aqueous solutions of (a) oxidized micro-graphite, (b) oxidized graphene nanoplatelets, (c) reduced graphene nanoplatelets and (d) graphene nanoribbons.

**Table 2:** EPR parameters of aqueous samples of oxidize micro-graphite, oxidized graphene nanoplatelets, reduced graphene nanoplatelets and graphene nanoribbons.

Sample	g-value	EPR Line width (ΔH_1/2_, Gauss) for g∼2.0	Hyperfine Coupling Constant A_Mn_, Gauss	Electron relaxation time(*T_2e_*, nanoseconds)
Oxidized micro-graphite	2.0067	29.2	94.5	2.25
Oxidized graphene nanoplatelets	2.0068	31.5	96.4	2.08
Reduced graphene nanoplatelets	2.0070	30.0	95.4	2.19
Graphene nanoribbons	2.0068	30.2	95.2	2.17

### 3. EPR Measurements

All the EPR spectra were measured at room temperature (∼296 K) under similar experimental conditions on a Bruker X-band EPR Spectrometer operating at ∼9.8 GHz microwave frequency with high 100 KHz magnetic field modulation frequency. The magnetic fields and g-values were calibrated with a standard solid sample of diphenyl picrylhydrazyl (DPPH, g = 2.0036). The EPR of blank quartz tube was measured to calibrate EPR baseline for the EPR spectra. All EPR spectra were measured twice, first with 1 k Gauss sweep width, and next with 6 k Gauss sweep width. The solid samples of graphite, graphene and controls were loaded into Wilmad Quartz EPR tubes. The quartz EPR sample tubes were washed thoroughly with deionized water, and dried prior to loading of the samples. The EPR measurements on the aqueous samples were done by using a quartz flat tube designed for aqueous and other solvents with high dielectric constants. Before loading the liquid samples, the quartz EPR flat tube was washed thoroughly with deionized water and dried. The loading of aqueous samples into the quartz flat tube was done carefully into the flat portion of the tube for maximum sensitivity.

**Table 3:** Relaxivity of oxidized graphite, oxidized graphene nanoplatelets, reduced graphene nanoplatelets and graphene nanoribbons dispersed in 1%Pluronic F127 solutions compared with clinically used MRI contrast agents.

Sample	*r_1_* (mM^−1^s^−1^)	*r_2_* (mM^−1^s^−1^)	*r_2_*/*r_1_*
Oxidized graphite	63 (61–78)	171 (169–184)	2.7
Oxidized Graphene nanoplatelets	52 (50–54)	114 (114–131)	2.2
Reduced graphene nanoplatelets	47 (34–49)	415 (389–430)	8.9
Graphene nanoribbons	62 (53–71)	303 (275–310)	4.9
Clinical Mn^2+^Chelate Complexes^ 30^	1.8–2.0	2.0–2.2	∼ 1
Clinical Gd^3+^Chelate Complexes^34^	3.4–5.8	3.6±7.0	∼ 1

### 4. Proton Relaxivity Measurements

For relaxivity measurements, 1 mg of oxidized micro-graphite, oxidized graphene nanoplatelets, reduced graphene nanoplatelets or graphene nanoribbon samples were dispersed in 2 ml of biologically compatible 1% Pluronic F127 surfactant solution, bath sonicated at 30 W for 10 min, and finally centrifuged at 5000 rpm for 1 h. The centrifugation allowed the non-water-solubilized large and dense graphene nanoparticles to settle to the bottom, and allowed the separation of soluble graphene nanoparticles in the supernatant. The supernatant solutions were also checked for the presence of any free Mn^2+^ ions. This was achieved by first flocculating the graphene nanoparticles with HCl, and then testing the clear solution with sodium bismuthate (NaBiO_3_) in HNO_3_. In this reaction, manganese is oxidized from the +2 oxidation state (Mn^+2^) to the +7 oxidation state (MnO_4_^-^) which has distinctive purple or pink color. No such color change was observed indicating that no free Mn^2+^ions (limit of detection 1 ppm or 1 µg/ml) were present in the supernatant solution.

**Figure 5:**
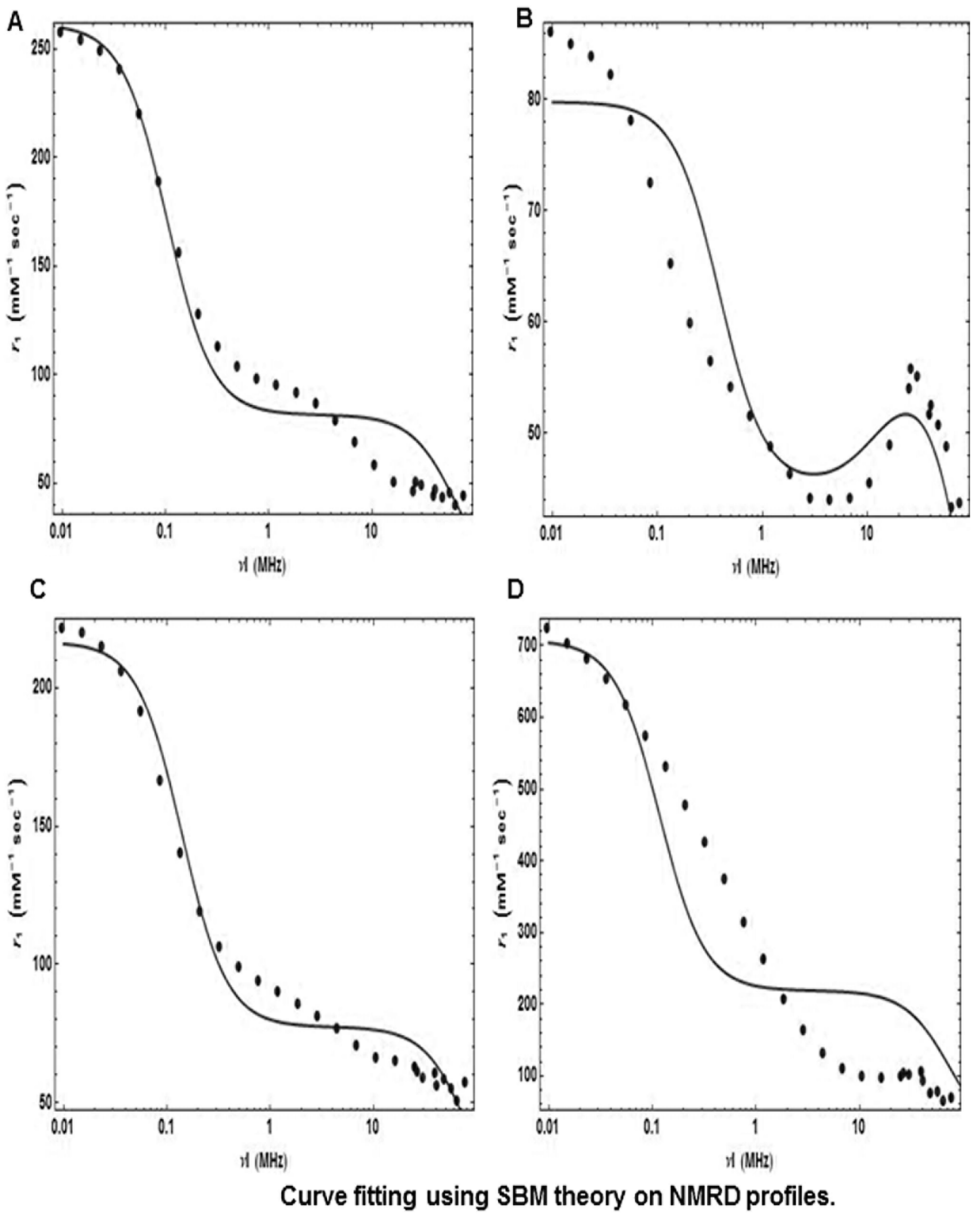
Experimental NMRD profiles (dots), and best fits (solid lines) derived from SBM Theory for (a) Oxidized Graphite, (b) Graphene Nanoplatelets, (c) Reduced Graphene Nanoplatelets, and d) Graphene Nanoribbons.

The supernatants solutions containing the soluble graphene nanoparticles were used for relaxometry measurements. The longitudinal and transverse relaxation times (*T*_1_, *T*_2_) were measured at 20 MHz (0.47 T) on a Minispec NMR spectrometer (Bruker Instruments, Woodland, Texas). Each sample was prepared at five known concentrations by serial dilution. The temperature was maintained at 40°C during the measurements. *T*_1_ and *T*_2_ relaxation times of each experimental sample and the control (1% Pluronic 127 solution) were measured using inversion recovery, and CPMG methods, respectively. The inverse of the relaxation times represent the respective relaxation rates, *R*_1_ and *R*_2_. A plot of relaxation rate (y-axis) versus concentration (x-axis) was created, and was fit to a linear curve. The slope of this linear fit gave the value of relaxivity. Single point relaxivity (*r_1_*) was obtained during NMRD measurements. The relaxivity values (*r_1_*), were calculated using the formula *r_1_* = (*R_1_*–*R_0_*)/[Mn^2+^]; where *R_1, 2_* and *R_0_* are the longitudinal or transverse relaxation rates of the samples, and 1% Pluronic F127 surfactant solution respectively, and [Mn^2+^] is the concentration of Manganese in the volume of solution used for relaxation measurements. The 1/*T_1_* NMR dispersion (NMRD) profiles at magnetic fields corresponding to a proton Larmor frequency range 0.01–40 MHz were obtained using a fast field cycling relaxometer (SPINMASTER FFC2000, Stelar Inc, Pavia, Italy). A High Field Superconducting Dipole (HTS) electromagnet was used to acquire the relaxation data from 25 to 80 MHz range of proton Larmor frequency. The temperature was fixed to 27°C, and was controlled by a Stelar VTC-91 airflow heater, equipped with a copper-constantan thermocouple; the temperature calibration in the probe head was done with a Delta OHM digital thermometer, with an absolute accuracy of 0.5°C.

**Table 4:** Computed parameters representing best fit to SBM equations.

Parameter	Definition	Oxidized Graphite	Oxidized Graphene Nanoplatelets	Reduced Graphene Nanoplatelets	Graphene Nanoribbons
Δ^2^	Zero-field splitting energy (ZFS)	1.0×10^18^	6.12×10^18^	1.0×10^18^	1.0×10^18^
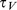 (sec)	Correlation time for splitting	1.18×10^−12^	1.09×10^−11^	1.99×10^−12^	1.0×10^−12^
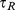 (sec)	Tumbling time of complex	1.95×10^−9^	1.77×10^−9^	3.85×10^−9^	3.69×10^−9^
*_q_*	Hydration number	8	8	8	8
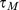 (sec)	Residence time ofinner sphere water molecules	1.42×10^−7^	7.29×10^−7^	7.06×10^−9^	5.06×10^−9^
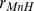 _(m)_	Manganese-Hydrogen Bond Radius	3.76×10^−10^	3.73×10^−10^	3.94×10^−10^	3.26×10^−10^

The aqueous graphene nanoplatelets and nanoribbons sample used for the relaxometry measurements were analyzed by Inductively-coupled plasma optical emission spectroscopy (ICP-OES) to determine the concentration of manganese. Additionally, iron content analysis was carried out for the aqueous graphene nanoribbons sample (see Text S1, table S1 and S2 for details).

**Table 5:** Relaxivity (*r_1_*) of Mn^2+^-based or Gd^3+^-based *T_1_* MRI contrast agents, and the dominant SBM parameter(s) that influence the relaxation mechanism.

Type of Compound	Mn^2+^-based		Gd^3+^-based		Parameter(s)
	Highest*r_1_* (mM^−1^s^−1^)	Magnetic field (MHz)	Highest*r_1_*(mM^−1^s^−1^)	Magnetic field (MHz)	
liposomal complex [3], [39]	35	20	11	25	*τ_M_*
Chelate complexes that non-covalent binding to Protein [3], [41], [45]	55	20	130	20	*τ_R_*
Dendrimer complex [45], [46]	4.7	200	20	130	*τ_R_*
Viral capsid complexes [5]	Not available	Not available	42	30	q,*τ_R_*
Small molecule complexes non-covalently functionalized to carbon nanotubes [7]	Not available	Not available	50	20	*τ_R_*
Small molecule complexes covalentlyfunctionalized to nano-diamonds [8]	Not available	Not available	59	60	*τ_R_*
Metallofullerenes [9]–[11]	Not available	Not available	8–100	20–50	q, *τ_R_*
Metallonanotubes [12], [13]	Not available	Not available	400–635	0.01	q, *τ_M_*_ ,_ *τ_R_*

## Results and Discussion

The structural, chemical and elemental analysis of oxidized graphite, oxidized graphene nanoplatelets, reduced graphene nanoplatelets and graphene nanoribbons are presented in the text S1 section 1 and 2 and Figure S1, S2, S3 and S4. Figure 1 shows the SQUID magnetic characterization of oxidized graphite, oxidized graphene nanoplatelets and reduced graphene nanoplatelets. Analytical grade micro-graphite used as the starting material for the preparation of these particles was the control in these experiments. Figure 1a shows the plot of magnetization (M) versus magnetic field strength (H) for the analytical grade micro-graphite (control) between −50,000 Oe and 50,000 Oe for three temperatures (30 K, 150 K, and 300 K). The negative slope indicates a decrease in the value of magnetic moments with increase in applied magnetic field, which is characteristic of diamagnetic behavior. Figure 1b and c shows the M versus H plot for oxidized graphite and oxidized graphene nanoplatelets, respectively. The plots show a linear increase in the value of the magnetic moments with field strength indicating paramagnetic behavior for both oxidized graphite and oxidized graphene nanoplatelets. The change to paramagnetism upon oxidation of graphite can be attributed to the presence of the paramagnetic Mn^2+^ ions present in the sample. Figure 1d shows the M versus H plot of reduced graphene nanoplatelets. The plot displays a ferromagnetic hysteresis curve at the lower temperature (30 K) indicating superparamagnetic behavior (inset of Figure 1d) at room temperature (300 K). Room temperature superparamagnetism has been widely reported in nanoparticle clusters (<30 nm) [23], [24], and is a size dependent phenomenon, wherein, the thermal energy of the nanoparticle is sufficient to allow flips in the magnetic spin direction, and insufficient to overcome the spin-spin exchange coupling energy. As a result, in the absence of a magnetic field, the net magnetization measured is zero, and the M versus H curve assumes an ‘S’ shape instead of a hysteresis loop. The zero field cooling (ZFC) and field cooling (FC) curves for the reduced graphene nanoplatelets at uniform field strength of 500 Oe and between 10 K and 300 K are shown in Figure 1e. The peak in the ZFC curve reveals a blocking temperature (T_B_) of 40 K indicating a transition between ferromagnetic and superparamagnetic states. The remnant magnetization of the hysteresis curve at 30 K is 12.47 emu/g and the coercivity is 6298.68 Oe and could be attributed to the single domain nature, and high shape anisotropy of the sample [25]. The results for reduced graphene nanoplatelets exhibit sharp resemblance with that of hausmannite [25]. Room temperature magnetism has been reported in carbon nanomaterials such as fullerenes, carbon nanotubes, carbon nanofoams, graphene, nanodiamonds and graphite [16], [17], [26]. The magnetic characteristic of these materials include spin-glass-like paramagnetic or ferromagnetic behavior attributed either to the presence of metal impurities or presence of defects in the graphite lattice structure. In case of the oxidized graphite, oxidized graphene nanoplatelets and reduced graphene nanoplatelets, the defects created in graphitic lattice structure during the oxidation or exfoliation process may contribute to the observed magnetic behavior. However, theoretical and experimental studies show the defects in graphitic structures induce very weak magnetic behavior with saturation magnetic moment values of approximately 10^−3^–10^−6^ emu/g [27]. Thus, the observed magnetic behavior reported above should be mainly due to the presence of manganese.

Figure 2 shows the SQUID magnetic characterization of MWCNTs (control), and graphene nanoribbons. Figure 2a shows the plot of magnetization (M) versus magnetic field strength (H) for the MWCNTs between −50,000 Oe and 50,000 Oe for three temperatures (10 K, 150 K, and 300 K). The plots show no coherent magnetic pattern, and the magnetic signals are extremely weak at all three temperatures indicating diamagnetic behavior despite the presence of iron catalysts in the MWCNTs.

Figure 2b displays the plot of M versus H for graphene nanoribbons between −50,000 Oe and 50,000 Oe for three temperatures (10 K, 150 K, and 300 K). Even though, the M versus H curve seems to assume an ‘S’ shape instead of a hysteresis loop, closer analysis of the curve (see inset in Figure 2b) indicates ferromagnetic behavior with a very low remanence. The SQUID analysis indicates ferromagnetism at 30 K, 150 K and 300 K. Closer analysis shows interesting magnetic properties at room temperature. The temperature dependence of the magnetization at zero-field cooled (ZFC) as well as field cooled (FC) conditions is plotted in Figure 2c at magnetic field strength 500 Oe (temperature range 10–300 K). It is clear from the graph that all the graphene nanoribbons show ferromagnetic behavior at low temperatures, and show bifurcation of the ZFC and FC branches. The temperature at which the FC and ZFC curves bifurcate (also referred as the irreversibility temperature), as well as the blocking temperature (T_B_) is 300 K. Figure 2c indicates FC/ZFC plots, and a maximum value on the ZFC curve is seen at a value >300 K, which is greater than room temperature. The ZFC magnetization curves show a broad maximum below the bifurcation temperature. The bifurcating FC and ZFC curves indicate thermodynamic irreversibility, and could have its origin in the effects like strong competing interaction between ferromagnetic and anti-ferromagnetic phases, and phase separations at a nanoscale due to the occurrence of a low temperature spin-glass-like state or a mixed phase [27]–[29]. The saturation magnetization seen at 300 K is 0.1 emu/g at 2500 Oe. The sample shows a coercive field of 250 Oe at 10 K. The magnetism results clearly indicate that the graphene nanoribbons exhibit room-temperature weak ferromagnetism. The elemental analysis of graphene nanoribbons showed that apart from manganese, trace amounts of iron (0.005 wt% or 50 µg Fe per gram, see table S1 and S2 for ICP analysis) was also present in these samples. The MWCNTs used in the preparation of the graphene nanoribbons do not show any magnetic behavior even though they contain iron nanoparticles as catalyst (0.1 wt%) [30] which is 20 times greater than the amount found in graphene nanoribbons. Furthermore, it has been reported that presence of Fe or Fe_3_O_4_ clusters with Fe concentration of 1–500 µg Fe per gram (1 ppm) graphite contribute 2.2×10^−5^ to 4×10^−3^ emu/g to the magnetization [26]. The above information taken together suggests that the presence of trace amounts of iron does not contribute significantly to the observed magnetic behavior of the graphene nanoribbons. Several recent studies show that point defects of oxygen vacancies in metal oxide nanostructures could result in weak ferromagnetism [31], [32], and similar defect in the manganese oxide due to its interactions with the graphene nanoribbons could be responsible for observed magnetic behavior. However, more studies are needed to confirm this hypothesis.

Figure 3a–d show the EPR spectra of the oxidized micro-graphite, oxidized graphene nanoplatelets, reduced graphene nanoplatelets and graphene nanoribbons, respectively (the blank EPR spectrum of the quartz EPR tube and DPPH standard is shown in in the Figure S5). The g values, EPR line widths at half heights (ΔH_1/2_, Gauss) and electron relaxation time (T_2e_) of each EPR spectra are listed in Table 1. All samples show broad peak (ΔH_1/2_) at their respective g values. However, graphene nanoribbons show ΔH_1/2_ values 2.6 times greater than oxidized micrographite, oxidized graphene nanoplatelets and reduced graphene nanoplatelets, which have similar ΔH_1/2_ values. The large line width indicates short electron relaxation time (*T_2e_*), and the calculated *T_2e_* values were between 0.19–21 nanoseconds for oxidized micrographite, oxidized graphene nanoplatelets, and reduced graphene nanoplatelets. Graphene nanoribbons have *T_2e_* values 0.072 nanoseconds; at least 2.9 times shorter than the other compounds. The EPR spectra of the graphene nanoribbons samples also shows a narrow peak in the center, which indicates presence of free radical species, possibly due to defect centers in the nanoribbon structures as reported by Tour et al. [33]. The free radical species have g of 2.007, and line width of 1.2 Gauss, and thus have very long electron relaxation time (*T_2e_*) of 88.2 nanoseconds. The large line broadening in all the compounds indicates significant manganese-to-manganese dipolar interaction. A reduction in the amount of manganese in the sample should decrease the line broadening, and resolve the 6-line manganese hyperfine structure in the EPR spectrum, and consequently, decrease the electron relaxation time.

Figure 4a–d show the EPR spectra of aqueous solutions of oxidized micro-graphite, oxidized graphene nanoplatelets, reduced graphene nanoplatelets and graphene nanoribbons, respectively (the blank EPR spectrum of the quartz EPR tube and the EPR spectrum of the DPPH is shown in in the Figure S5). The g values, EPR line widths at half heights (ΔH_1/2_, Gauss), hyperfine coupling constant, and electron relaxation time (T_2e_) of each EPR spectra are listed in Table 2. All the four samples show 6-line EPR characteristic of an electron coupled to Mn-55 nucleus with spin I = 5/2. The EPR spectra of graphene nanoribbons also show a narrow EPR line at the center with g∼2.007, and line width of 1.2 Gauss due to the presence of free radicals. The observed *g* values are very close to the free electron spin value, and suggest the absence of spin-orbit coupling in the ground state of manganese ions present in all four samples. The manganese hyperfine coupling (A_Mn_) of approximately 95 Gauss in these samples are very close to that of aqua ions of manganese, Mn(H_2_O)_6_. The large hyperfine coupling indicates octahedral coordination in the manganese species of all four samples. The four aqueous samples also show similar narrow line width (ΔH_1/2_) values between 29.2–31.5 Gauss indicative of long electron relaxation time (*T_2e_*). The calculated *T_2e_* values were between 2.08–2.25 ns. The free radical species present in the graphene nanoribbons have an order of magnitude longer electron relaxation time (*T_2e_*) of 55 ns. It should be noted that the EPR spectra only shows the Mn(II) ions. The spectra did not show presence of Mn(III) ions or other oxidation states of manganese even though, the Raman spectrum of at least reduced graphene nanoplatelets show the presence of Mn(III) ions. A possible reason of this non-detection could be that all the EPR measurements were done at room temperature. Mn(III) ions or other oxidation states of Manganese have very short electron relaxation times, and require very low sample temperatures (∼77 K) to obtain an EPR spectra. Thus, low temperature measurements were also carried out on all the four samples. However, the EPR spectra (results not shown) was dominated by Mn (II) contributions, and the presence of other oxidation states of manganese could not be confirmed, suggesting that most of the manganese ions present in the four samples are present in Mn(II) state.

Relaxivity (*r_1, 2_*) is an important measure of the efficacy of an MRI contrast agent. Table 3 shows the relaxivity values at 0.47 T for oxidized micro-graphite, oxidized graphene nanoplatelets, reduced graphene nanoplatelets and graphene nanoribbons at 40°C. Also included for comparative purposes are range of relaxivity values of clinically approved Gd^3+^-based and Mn^2+^ based chelate complexes [34]. The table clearly shows that all four compounds show significantly higher *r_1_* and *r_2_* relaxivities compared to paramagnetic chelate complexes. At 0.47 T, the *r_1_* and *r_2_* values for the graphite and graphene samples are ∼8–10 times, and 19–60 times greater than paramagnetic chelate complexes. Among the graphitic and graphene samples, at 0.47 T, graphene nanoribbons, and oxidized graphite showed higher (∼20%) *r_1_* values than oxidized graphene nanoplatelets and reduced graphene nanoplatelets. However, the trend for *r_2_*:*r_1_* ratio was reduced graphene nanoplatelets >graphene nanoribbons >oxidized micro-graphite >oxidized graphene nanoplatelets. This trend is along expected lines since, the magnetism results show that graphene nanoplatelets and graphene nanoribbons are superparamagnetic at 40°C. It well-known that superparamagnetic materials mainly affect transverse *T_2_* relaxation and thus, increase the *r_2_*/*r_1_* ratio. However the *r_2_*/*r_1_* ratio is lower than iron-based *T_2_* contrast agents that have ratios of 10 or more. *T_1_* contrast agents have *r_2_*/*r_1_* ratios about 1∼2 [35]. Thus, the manganese-intercalated graphitic, and graphene particles may be better suited as *T_1_* contrast agents even though at higher fields (3 T or above), the reduced graphene nanoplatelets and graphene nanoribbons would give rise to T_2_* effects. In case of the graphene nanoribbons, iron (the catalyst used to prepare the MWCNTs) could not be detected (limit of detection 1 ppb) in the aqueous samples used for relaxivity measurements (see table S2 ICP analysis). *T_1_* relaxation measurements at 60 MHz on 1 ppb iron chloride solution showed that the presence of iron at this low concentration show negligible change (within the error of the instrument) in the *T_1_* relaxation time compared to deionized water. Additionally, relaxivity measurements at 60 MHz of metal free (no paramagnetic ions) graphene solutions (unpublished results) also indicate that the presence of free radicals do not affect the relaxation time. Thus, in this study, for the graphene nanoribbons, the presence of additional components such a metal catalysts or free radicals do not shorten the *T_1_* relaxation. However, it should be mentioned higher amounts of iron catalyst (in the ppm range) could potentially confound the relaxivity values, and the interpretation of the NMRD data, and thus, extra precaution should be taken during the preparation, and purification of the graphene nanoribbons to ensure the complete removal of the iron catalyst.

The NMRD profiles between 0.01–80 MHz of aqueous solutions of oxidized graphite, oxidized graphene nanoplatelets, reduced graphene nanoplatelets and graphene nanoribbons is presented in Figure 5a–d. This is the first report of longitudinal *r_1_* relaxivities for these compounds over such a large magnetic field range (0.01–80). While oxidized micro-graphite and reduced graphene nanoplatelets show similar NMRD profiles, oxidized graphene nanoplatelets, and graphene nanoribbons show distinctly different profiles than these two samples. At mid-to-high magnetic field (<10 MHz), oxidized micro-graphite shows a smaller increase (50–66 mM^−1^s^−1^) with decrease in magnetic field, and a greater increase with decrease to lower magnetic fields (70–222 mM^−1^s^−1^). Oxidized graphene nanoplatelets shows bell shaped distribution at mid-to-high magnetic fields with a maximum of 55 mM^−1^s^−1^ at 30 MHz, and a gradual increase up to 86 mM^1^s^−1^ as the magnetic fields decrease below 10 MHz. Reduced graphene nanoplatelets shows a small increase (44–59 mM^−1^s^−1^) with decrease in magnetic field between 80–10 MHz, and the relaxivity increases at lower magnetic fields with a maximum value of 258 mM^−1^s^−1^ at 0.01 MHz. Graphene nanoribbons show a linear increase (relaxivity between 65–100 mM^−1^s^−1^) with decrease in magnetic fields up to 10 MHz, and then a continuous steep increase below 10 MHz reaching values of 724 mM^−1^s^−1^ at 0.01 MHz.

The NMRD profiles of these compounds are different than the profiles of other manganese-based small molecular or macromolecular complexes [3], [36]. For example, small molecule Mn^2+^ complexes such as Mn-DTPA (DTPA = diethylene triamine penta-acetic acid) show a constant values of ∼1.9 mM^−1^s^−1^ at fields greater than 10 MHz, and marginal increase at fields less than 10 MHz. Macromolecular complexes Mn^2+^-DTPA-BSA (BSA = bovine serum albumin) show a bell-shaped relaxivity distribution at magnetic field between 10–80 MHz with a peak value of 26 mM^−1^s^−1^ at 20 MHz [3]. At magnetic fields less than 10 MHz, the relaxivity is constant at ∼14 mM^−1^s^−1^. Similar profiles have been reported for small and large molecule complexes of Gd^3+^ ions [3]. The profiles are also different than profiles of Gd3+@C60 (gadofullerenes) which show profiles similar to those of Mn^2+^- or Gd^3+^ macromolecular complexes [9]. However, the profiles of Gd3+@ultrashort-single-walled carbon tubes (gadonanotubes) [13] have features similar to those observed by Mn^2+^ intercalated graphitic and graphene compounds, i.e. increase in relaxivity with decrease in magnetic field with a greater increase at magnetic fields below 10 MHz. The profile of the gadonanotubes at lower magnetic fields (<10 MHz) is most similar to that of graphene nanoribbons.

The Solomon-Bloembergan-Morgan (SBM) set of equations (see text S1, section 3) are considered to give the best theoretical description on how factors such as the water proton interactions with the contrast agent, magnetic properties of the contrast agent, and the molecular dynamics of the contrast agent affect the relaxation rate of the water protons at magnetic fields greater than 0.1 Tesla [4]. It is widely accepted that there are three types of water molecules that can be influenced by the MRI CA: (a) the water molecules directly co-ordinated to the paramagnetic metal center of the CA are known as the inner-sphere water molecules; (b) the water molecules not co-ordinated to the magnetic metal center of the contrast agent, but chemically-bound to other molecules (e.g. ligands, chelates) of the CA are called the second sphere water molecules; and (c) the more distant water molecules that are not bound to the MRI CA, but diffuse close to it are termed the outer-sphere water molecules. Experimental nuclear magnetic relaxation dispersion (NMRD) profiles are typically fit using the SBM equations to determine these factors that influence proton relaxivity [1]–[4]. Recent reports suggest that for gadonanotubes, the factors that govern their interactions with the inner-sphere water protons such as proton/water exchange rate, and the rotational correlation time are responsible for most of the observed *r_1_* relaxivity [13]. Thus, we have mainly focused on SBM equations that describe the inner-sphere interactions. Figure 5a–d show the NMRD profiles of the oxidized graphite**,** oxidized graphene nanoplatelets, reduced graphene nanoplatelets, and graphene nanoribbons, respectively. Also included are the corresponding best-fit, physically reasonable values (within the range of values reported for other Gd(III) and Mn(II)-based compounds) for the various inner-sphere parameters. (A discussion of our fitting approach is presented in the text S1, section 3, Figures S6,S7,S8,S9,S10 S11 and tables S4,S5,S6,S7,S8,S9). Table 4 lists the computed parameters, their definitions and values (table S3 lists the fixed parameters, their definitions and values). In general, the SBM equations provide an acceptable fit at high fields (>10 MHz) or low field (<0.5 MHz). Overall, the fits were more accurate for oxidized micro-graphite, and reduced graphene nanoplatelets than for oxidized graphene nanoplatelets and nanoribbons. This indicates that the SBM equations may not be an entirely satisfactory model for all the compounds synthesized here. Nevertheless, below we discuss the parameters returned by the curve-fitting algorithm to examine if they are in line with those reported elsewhere.

The parameter 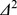 represents the zero-field splitting energy of the paramagnetic metal’s electrons. Even in the absence of an applied field, which is normally used to produce Zeeman splitting, splitting can still occur due to random motions and distortions of the complex. The fields generated by these interactions produce energy which induces relaxation in the nearby protons. The correlation time for this splitting is termed 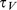. These two parameters are important in determining the effectiveness of the paramagnetic center. 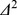 is generally in the range of 10^18^–10^20^ s^−2^. The values found from the fits are well within the accepted range. The value of 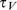 is generally accepted as being from 1–100 picoseconds [3]. The values we have found are in this range. In case of 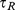, the rotational correlation time, Aime [37], Lauffer [3], and Toth [9] *et al* report values in the 10 ps to 2 ns range, while Ananta [13] reports that gadonanotubes can have values dropping into the nanosecond to microsecond range. The results obtained for the micro-graphite and graphene samples are in the nanosecond time scale. The parameter 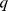 represents the number of fast-exchanging water molecules within the inner sphere, and its value was 8 for all the samples. These values fall outside the range of values for 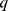 obtained for various paramagnetic complexes, which are between 1 and 6._ENREF_35 However, *q* values as high as high as 20 have reported for gadofullerenes [9]. Theoretical studies on Manganese intercalation within graphene suggest coordination of manganese to the graphene sheets with 1–3 co-ordination bonds [38]. Assuming most of the intercalated graphene is Mn^2+^ in the high spin state, the co-ordination number can be between 4 and 8 and thus, the possible co-ordination sites for water molecules will be between 1 and 7, and value obtained from the NMRD fits is close to this value. Additionally, the EPR results also indicate that this value is reasonable. The parameter 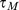, the water-residence lifetime has a dual effect on the relaxivity. On one hand, the longer a water molecule is resident in the inner sphere, the more time the paramagnetic center can influence its spin. However, if its resident time is too long, it blocks the ability of other water molecules from co-ordinating to the paramagnetic metal center, and can reduce the overall relaxivity. Hence, the optimum relaxivity is somewhere between the possible extremes. Literature reports show a wide range 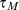 values. Small molecule complexes are generally in the range of 11–100 ps, while macromolecules such as paramagnetic liposomes [39], gadofullerenes [9], gadonanotubes [13] have values between 100–500 ns. The values found from the fits range between a few to hundreds of nanoseconds. To corroborate this data, ^17^O measurements were performed at 14 T, and the water exchange correlation time (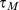) was estimated by analyzing the data according to the Swift and Connick theory (see text S1, section 4) [40]. The 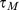 value was estimated to be hundreds of ns for all samples at 27°C. While these values corroborate well with the 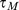 values obtained from NMRD fits oxidized micro-graphite and oxidized graphene nanoplatelets, they are 100 times greater than the values of reduced graphene nanoplatelets and graphene nanoribbons. The NMRD fits obtained by fixing the values of 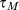 at hundreds of nanoseconds for these two samples gave good fits, and reasonable values for other parameters in case of reduced graphene nanoplatelets, however, a poor fit was obtained for graphene nanoribbons (See Figure S11). The separation distance, 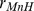 between the water protons and the paramagnetic metal ion (Mn^2+^ ion in this case) is raised to the 6^th^ power in the SBM equations. Thus, it has a very large influence on relaxivity, with shorter the distance, larger the influence. In this work, we found that allowing the parameter to vary slightly, rather than hold it fixed at the most commonly reported value of 2.9 angstroms [41]. The fitting values we obtained were in any case very close to the nominal value, but due to the extreme sensitivity of the SBM equations toward this value, it allowed for improved fits.

Multiple approaches have been developed wherein the above factors that affect the relaxation mechanism have been altered to design new high-efficiency Mn^2+^-based or Gd^3+^-based *T*_1_ MRI CA (Table 5). These approaches have focused on altering one or more of the following parameters: (1) increasing the number of inner-sphere water molecules (*q*); (2) decreasing the inner-sphere water residence lifetime (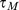), and increasing the rotational correlation time (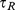) of the contrast agent (CA); (3) decreasing the *r_MnH_* by altering bond angles and orientation when designing chelates [42]. In the case of Mn^2+^ based macromolecular contrast agents, at 20 MHz, *r_1_* values as high as 55 mM^−1^ have been reported compared to Mn^2+^ ions without any chelate or chelated with various small molecule polycarboxylic acid ligands which show *r_1_* values between 4–10 mM^−1^s^−1^. The two parameters that have been manipulated in these studies are 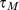 and/or 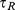. The results of this work introduce a novel general approach to enhance the *r_1_* relaxivity by confining the paramagnetic metal between graphene sheets, allowing the characteristic parameters *q*,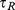, and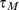 to be modified accordingly. The results indicate that confinement (intercalation) of paramagnetic metal ions within graphene sheets, and not the size, shape or architecture of the graphitic carbon particles is the key determinant for increasing relaxivity, and thus, identifies nano confinement of paramagnetic ions as novel general strategy to develop metal-ion graphitic-carbon complexes as high relaxivity MRI CA.

The physiochemical characterization, and the promising relaxivity results of the graphitic, and graphene structures reported in the work opens avenues for *in vitro* and *in vivo* studies to assess their safety and efficacy as MRI CAs. According to a recent report, in the US, approximately 43% of the 27.5 million clinical MRI procedures use CAs and the MRI CA market is projected to grow to $1.87 billion in 2012 [43]. Most clinical MRI CAs are gadolinium-(Gd^3+^) ion-based *T*_1_ paramagnetic CAs, that enhance MR signals to generate bright positive contrast. The recent discovery of nephrogenic systemic fibrosis (NSF) in some patients with severe renal disease or following liver transplant has generated concern leading to Food and Drug Administration (FDA) restrictions on clinical use of the Gd^3+^- ion based MRI CA [44]. Manganese, which was reported early on as an example of paramagnetic contrast material for MRI, has again received attention as a possible alternative to gadolinium [45]. Unlike the lanthanides, it is a natural cellular constituent resembling Ca^2+^, and often functions as a regulatory cofactor for enzymes and receptors. Normal daily dietary requirement for manganese is 0.1–0.4 milligrams, while normal serum levels are 1 nano-molar. Manganese toxicity has only been reported following long-term exposure or at high concentrations resulting in neurological symptoms [45]. Thus, further development of the micro- and nano-particles reported in this work could lead to development of a new class of Mn^2+^-carbon nanostructure complexes as high-efficacy MRI CAs.

## Supporting Information

Figure S1 **Representative SEM image of (a) oxidized micro-graphite and TEM images of (b,c) reduced graphene nanoplatelets and (d,e) graphene nanoribbons.**
(TIF)

Figure S2 **Representative TEM and AFM images. Arrows in (a) show the multiple layers of graphene nanoribbon sheets.** (b) TEM images at 200 kV for reduced graphene nanoplatelets Shows ∼20 nm wide few layered and multilayered reduced graphene nanoplatelets. (c) AFM Section analysis of graphene nanoplatelets dispersed on silicon substrate, showing a uniform thickness of ∼1.137 nm.
(TIF)

Figure S3 **Raman spectrum with the D and G bands peaks for (a) graphite, oxidized graphite, oxidized graphene nanoplatelets and reduced graphene nanoplatelets, and (b) MWCNTs and graphene nanoribbons (c) Comparison of Raman spectra between Hausmannite (Mn_3_O_4_), oxidized graphite and reduced graphene nanoplatelets at 532 nm showing spectral peaks at 657, 370 and 320 cm^−1^.**
(TIF)

Figure S4 **EELS spectrum for (a) reduced graphene nanoplatelets and (b) oxidized graphene nanoplatelets showing a oxygen peak at 530 eV.**
(TIF)

Figure S5 **EPR spectrum of the (a) Wilmad quartz EPR tubes used for the measurement of the solid samples, (b) quartz EPR flat tube used for the aqueous samples, (c) DPPH standard (solid) and (d) DPPH standard (aqueous).**
(TIF)

Figure S6 **Curves obtained with all SBM parameters floating.** A) Oxidized Graphite, B) Oxidized Graphene Nanoplatelets, C) Reduced Graphene Nanoplatelets, D) Graphene Nanoribbons.
(TIF)

Figure S7 **Curves obtained for fixed Q = 2 with remaining SBM parameters allowed to float.** A) Oxidized Graphite, B) Oxidized Graphene Nanoplatelets, C) Reduced Graphene Nanoplatelets, D) Graphene Nanoribbons.
(TIF)

Figure S8 **Curves obtained for fixed Q = 4 with remaining SBM parameters allowed to float.** A) Oxidized Graphite, B) Oxidized Graphene Nanoplatelets, C) Reduced Graphene Nanoplatelets, D) Graphene Nanoribbons.
(TIF)

Figure S9 **Curves obtained for fixed Q = 6 with remaining SBM parameters allowed to float.** A) Oxidized Graphite, B) Oxidized Graphene Nanoplatelets, C) Reduced Graphene Nanoplatelets, D) Graphene Nanoribbons.
(TIF)

Figure S10 **Curves obtained for fixed Q = 8 with remaining SBM parameters allowed to float.** A) Oxidized Graphite, B) Oxidized Graphene Nanoplatelets, C) Reduced Graphene Nanoplatelets, D) Graphene Nanoribbons.
(TIF)

Figure S11 **Curves obtained for fixed Q = 8 and Fixed T_m_ at values shown in Table S3, with remaining SBM parameters allowed to float.** A) Oxidized Graphite, B) Oxidized Graphene Nanoplatelets, C) Reduced Graphene Nanoplatelets, D) Graphene Nanoribbons. The fit for the Graphene Nanoribbons in D is surprisingly worse than expected.
(TIF)

Table S1 **Trace elemental analysis of solid samples of the oxidize micro-graphite, oxidized graphene nanoplatelets, reduced graphene nanoplatelets and graphene nanoribbons.** The standard deviation among the various batches was 10%.
(DOCX)

Table S2 **Trace elemental analysis of aqueous samples of the oxidized micrographite, oxidized graphene nanoplatelets, reduced graphene nanoplatelets and graphene nanoribbons.** The values presented are for one batch of samples.
(DOCX)

Table S3 **List of parameter values in SBM equations that are fixed constants, or independently established physical quantities.**
(DOCX)

Table S4 **SBM Parameters obtained from the curve fit with all parameter values floating.**
(DOCX)

Table S5 **SBM Parameters obtained from the curve fit for fixed Q = 2 and remaining SBM parameters allowed to float.**
(DOCX)

Table S6 **SBM Parameters obtained from the curve fit for fixed Q = 4 and remaining SBM parameters allowed to float.**
(DOCX)

Table S7 **SBM Parameters obtained from the curve fit for fixed Q = 6 and remaining SBM parameters allowed to float.**
(DOCX)

Table S8 **SBM Parameters obtained from the curve fit for fixed Q = 8 and remaining SBM parameters allowed to float.**
(DOCX)

Table S9 **SBM Parameters used to obtain curve fit for fixed Q = 8 and fixed Tm values.**
(DOCX)

Text S1 **Information on structural characterization, elemental and Raman analysis, Solomon-Bloembergan-Morgan Theory of Relaxivity and ^17^O-transverse relaxation rate measurements on oxidized micro graphite, graphene nanoplatelet and nanoribbons.**
(DOCX)
