# Supplementary figures and images for: Physicochemical Characterization, and Relaxometry Studies of Micro-Graphite Oxide, Graphene Nanoplatelets, and Nanoribbons
Source: PLoS One. 2012 Jun 7;7(6):e38185. doi: 10.1371/journal.pone.0038185 (PMC3369907; doi:10.1371/journal.pone.0038185)

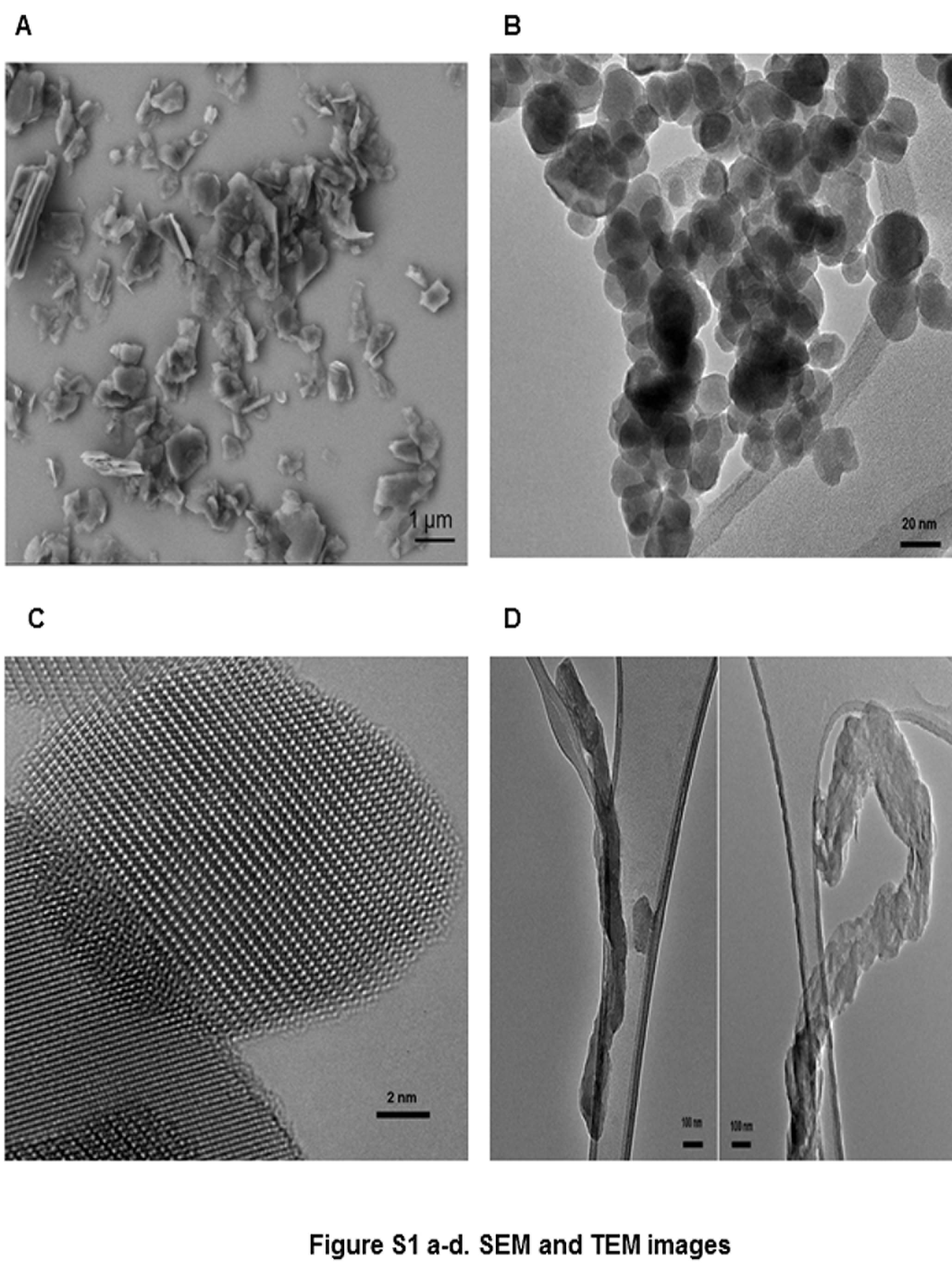

Supplement: Figure S1 — Representative SEM image of (a) oxidized micro-graphite and TEM images of (b,c) reduced graphene nanoplatelets and (d,e) graphene nanoribbons. (TIF) [file pone.0038185.s001.tif]

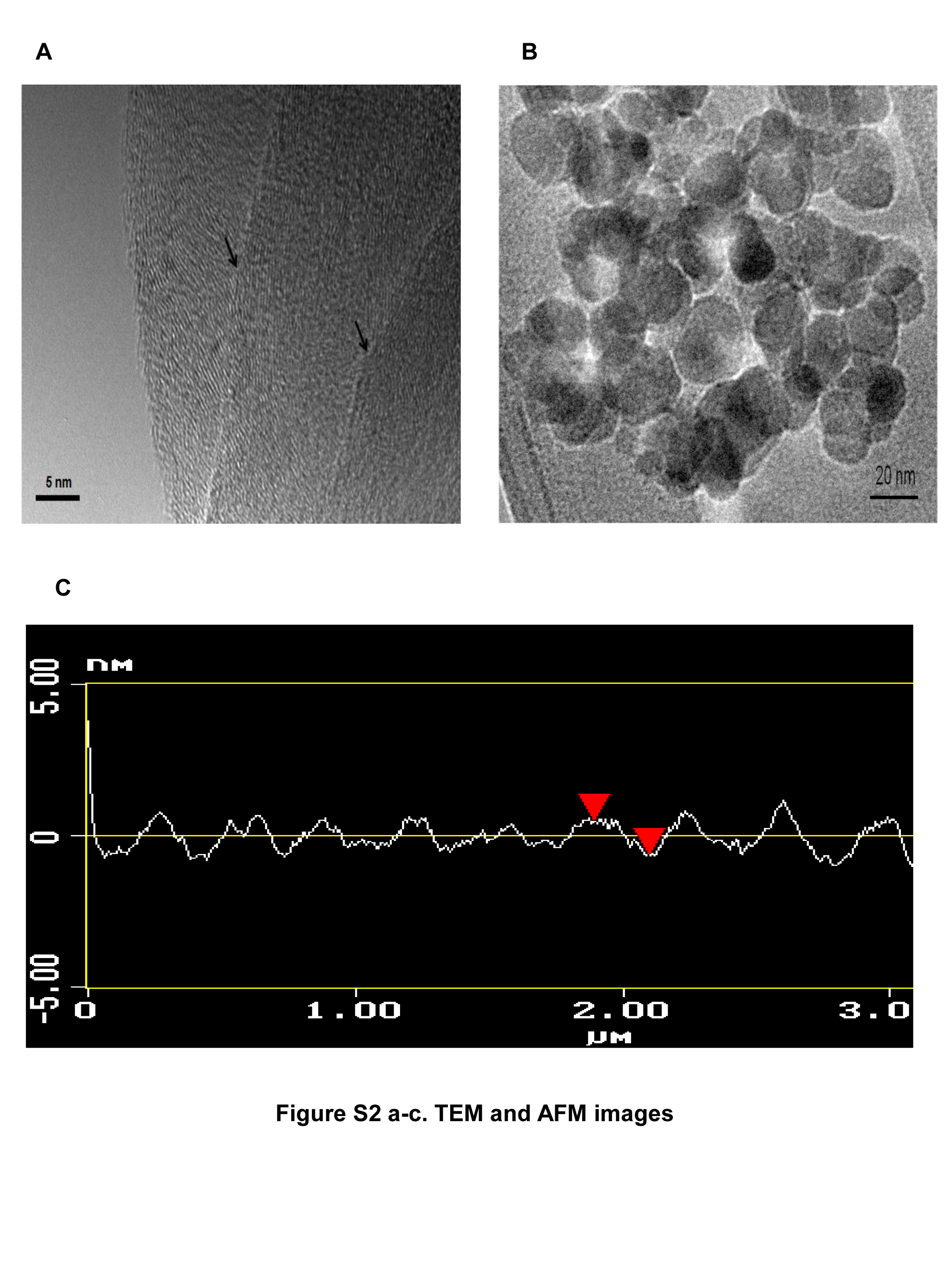

Supplement: Figure S2 — Representative TEM and AFM images. Arrows in (a) show the multiple layers of graphene nanoribbon sheets. (b) TEM images at 200 kV for reduced graphene nanoplatelets Shows ∼20 nm wide few layered and multilayered reduced graphene nanoplatelets. (c) AFM Section analysis of graphene nanoplatelets dispersed on silicon substrate, showing a uniform thickness of ∼1.137 nm. (TIF) [file pone.0038185.s002.tif]

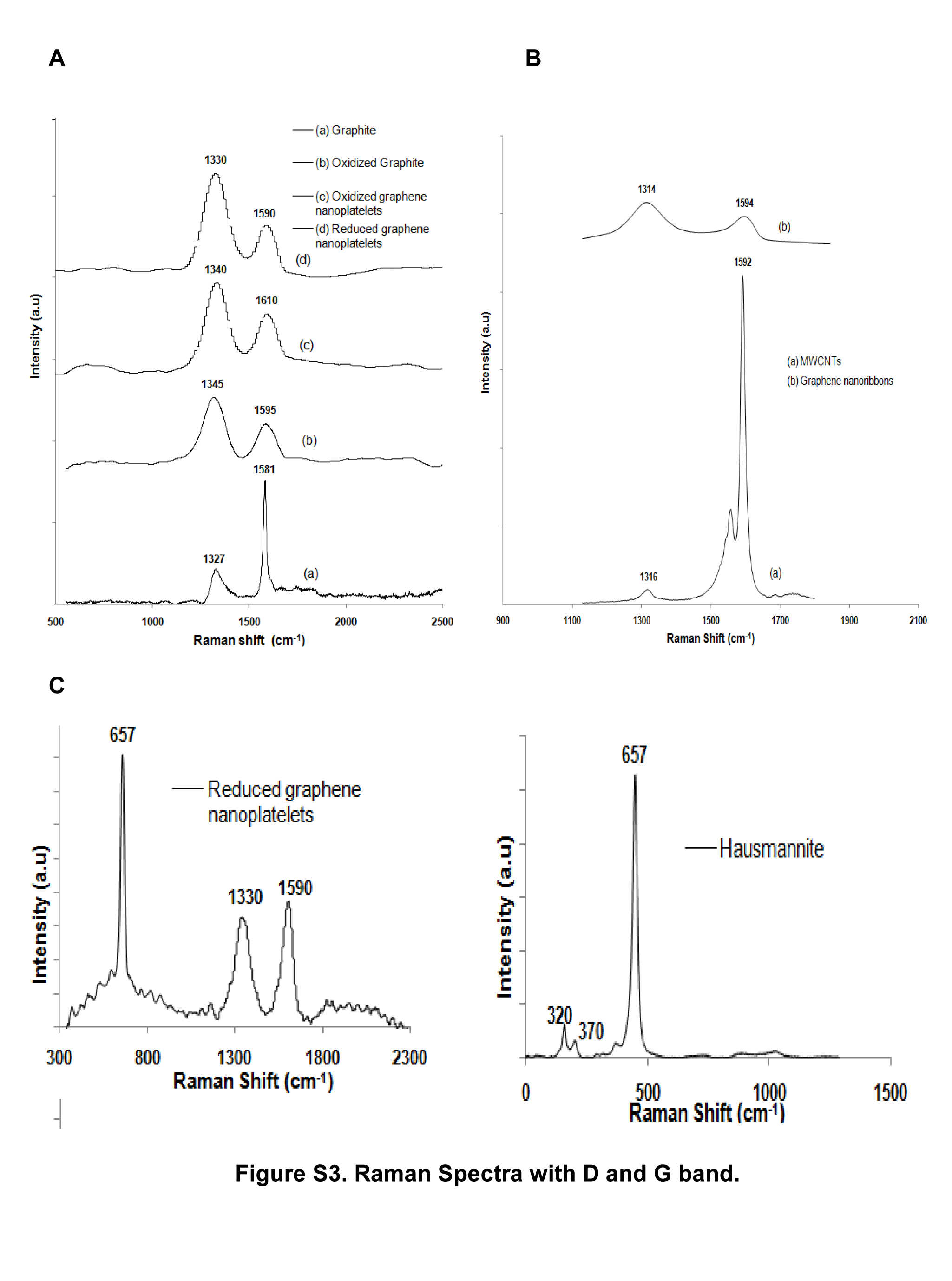

Supplement: Figure S3 — Raman spectrum with the D and G bands peaks for (a) graphite, oxidized graphite, oxidized graphene nanoplatelets and reduced graphene nanoplatelets, and (b) MWCNTs and graphene nanoribbons (c) Comparison of Raman spectra between Hausmannite (Mn3O4), oxidized graphite and reduced graphene nanoplatelets at 532 nm showing spectral peaks at 657, 370 and 320 cm−1. (TIF) [file pone.0038185.s003.tif]

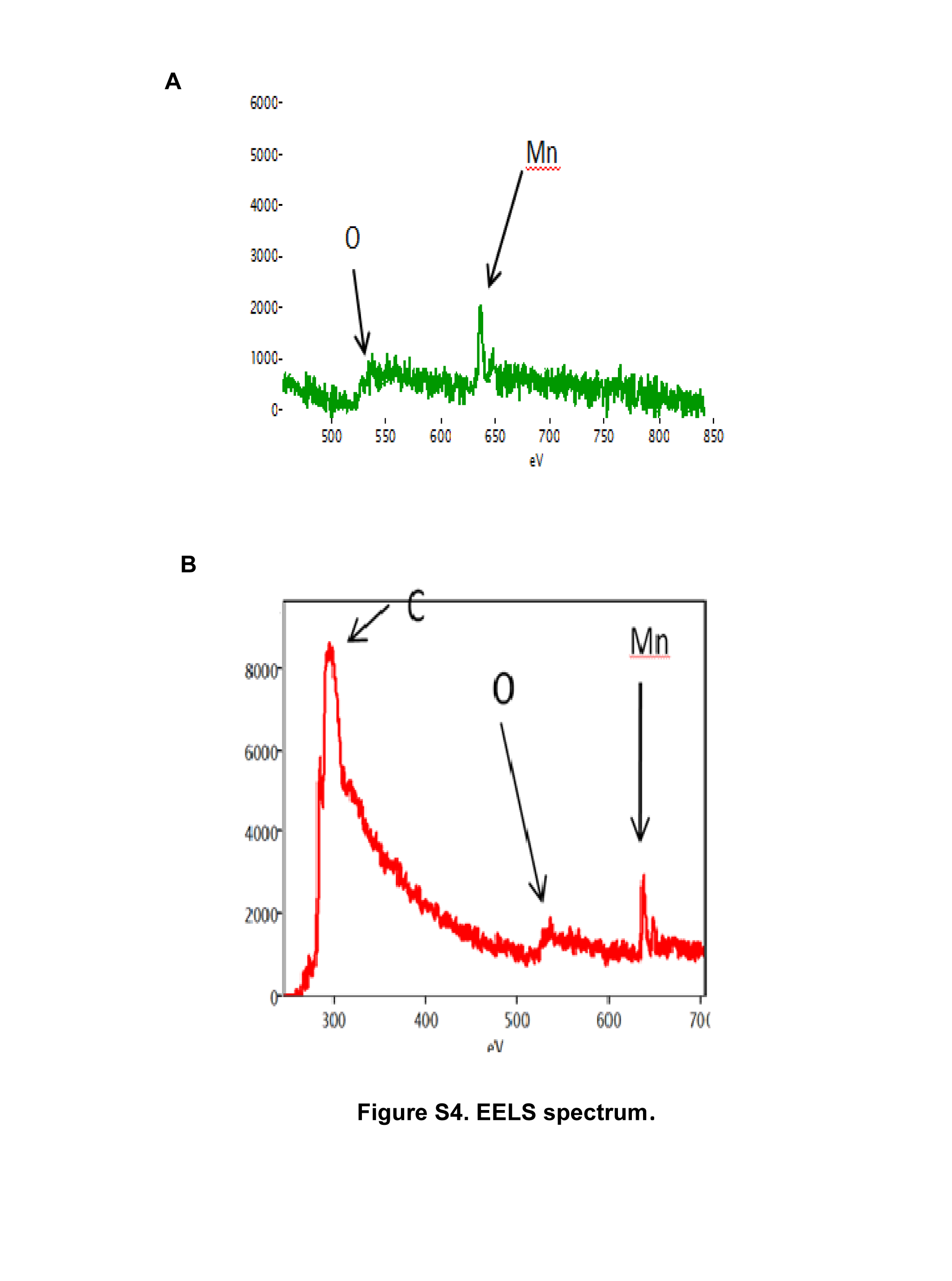

Supplement: Figure S4 — EELS spectrum for (a) reduced graphene nanoplatelets and (b) oxidized graphene nanoplatelets showing a oxygen peak at 530 eV. (TIF) [file pone.0038185.s004.tif]

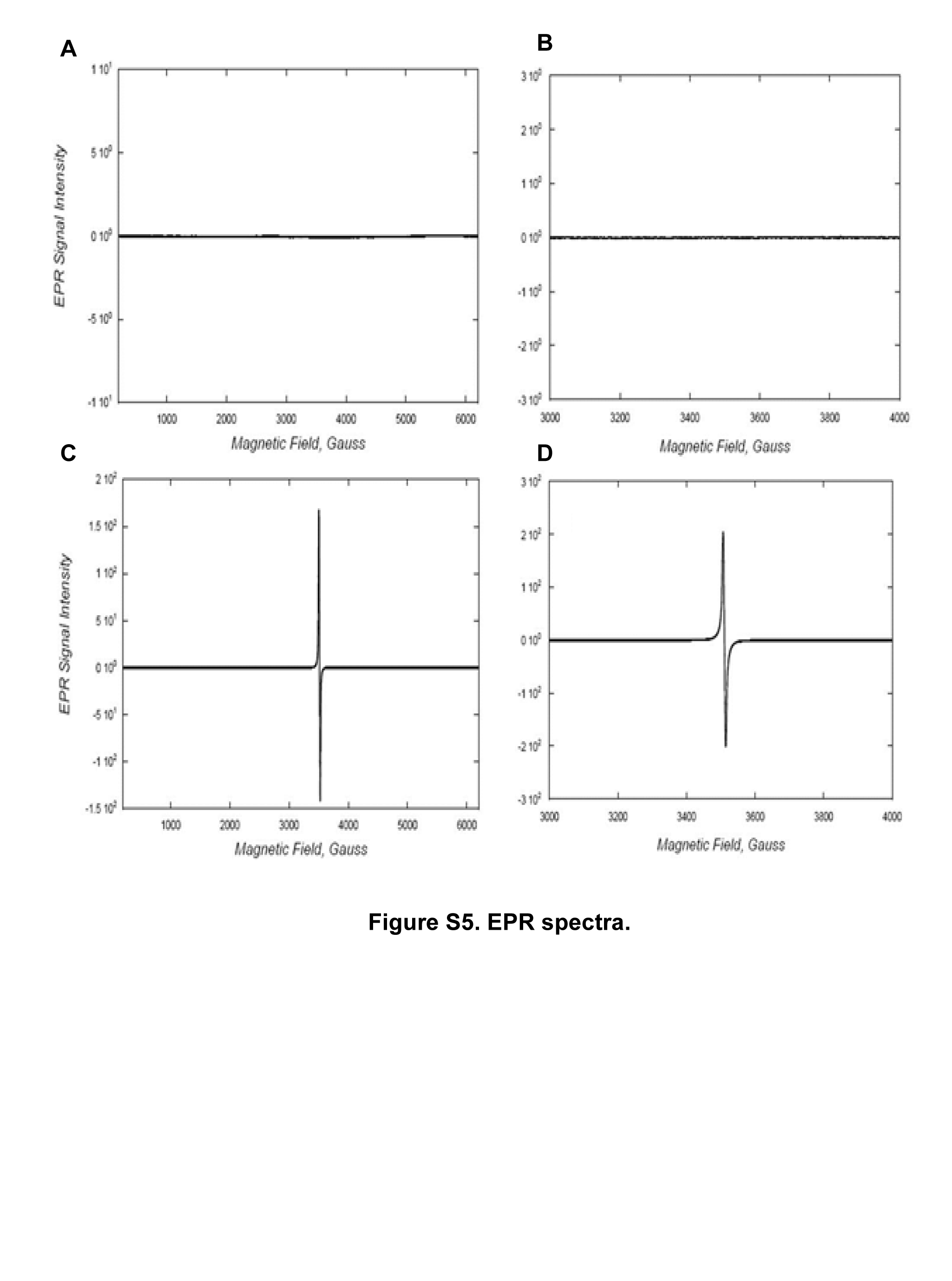

Supplement: Figure S5 — EPR spectrum of the (a) Wilmad quartz EPR tubes used for the measurement of the solid samples, (b) quartz EPR flat tube used for the aqueous samples, (c) DPPH standard (solid) and (d) DPPH standard (aqueous). (TIF) [file pone.0038185.s005.tif]

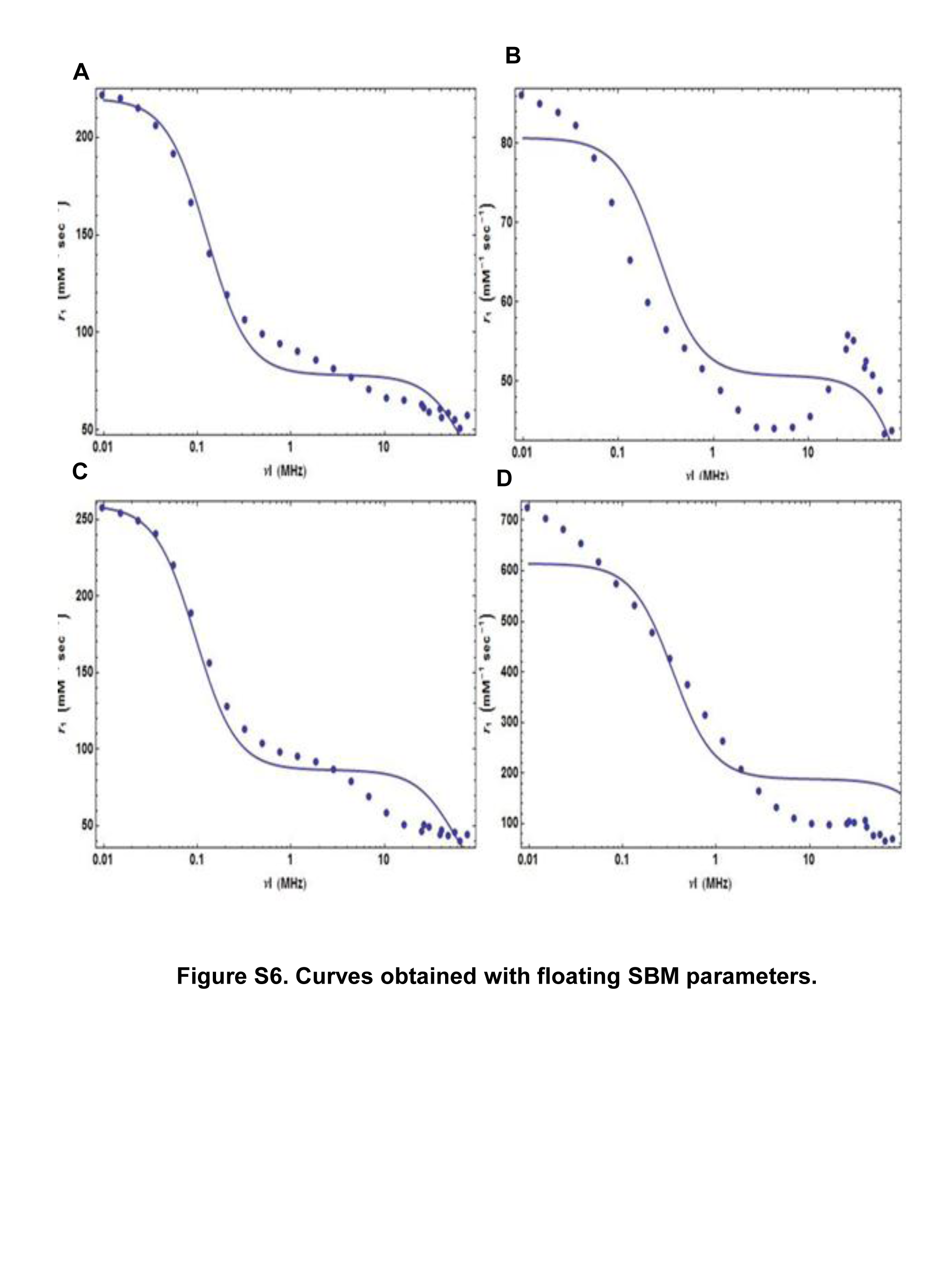

Supplement: Figure S6 — Curves obtained with all SBM parameters floating. A) Oxidized Graphite, B) Oxidized Graphene Nanoplatelets, C) Reduced Graphene Nanoplatelets, D) Graphene Nanoribbons. (TIF) [file pone.0038185.s006.tif]

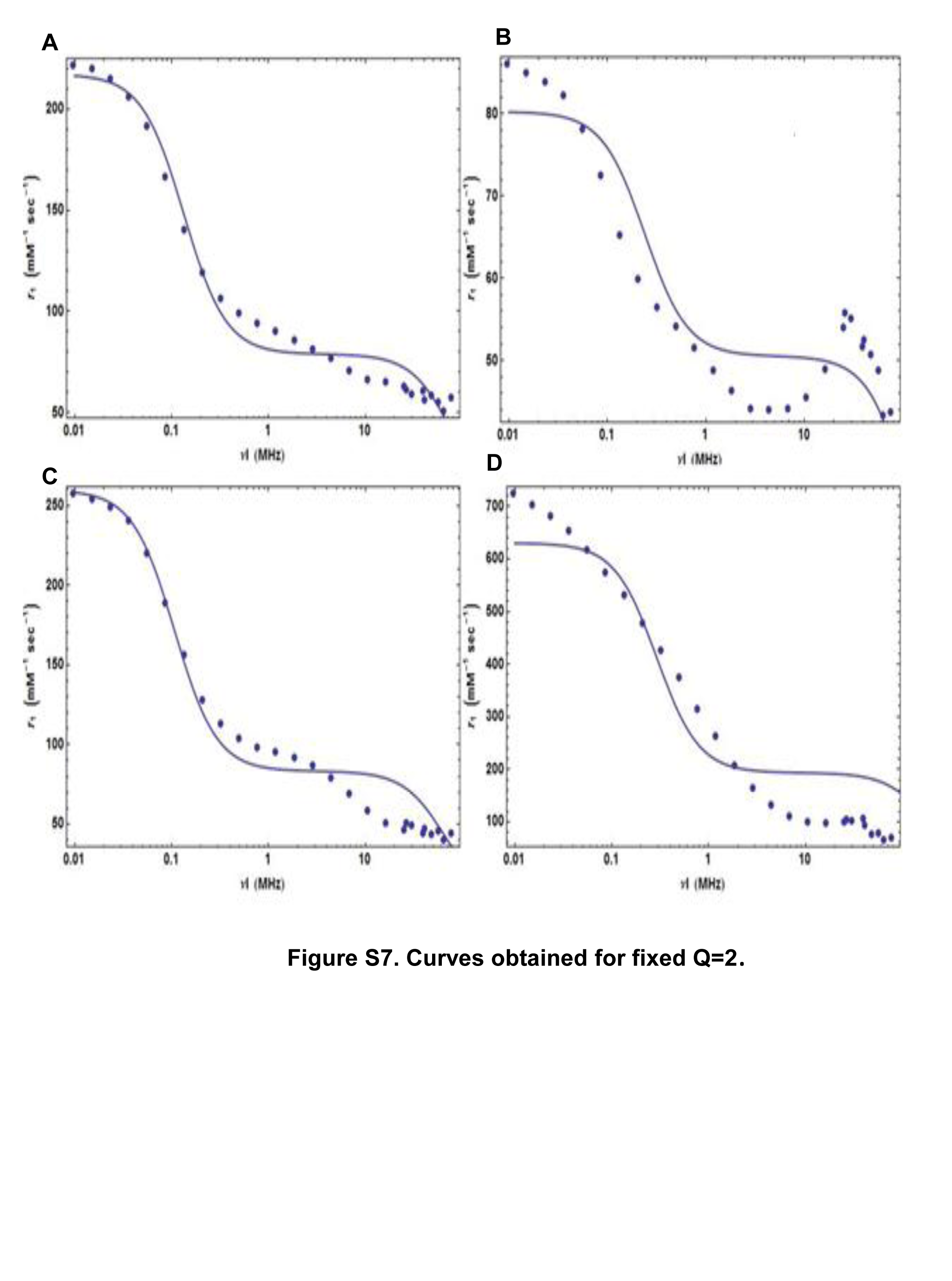

Supplement: Figure S7 — Curves obtained for fixed Q = 2 with remaining SBM parameters allowed to float. A) Oxidized Graphite, B) Oxidized Graphene Nanoplatelets, C) Reduced Graphene Nanoplatelets, D) Graphene Nanoribbons. (TIF) [file pone.0038185.s007.tif]

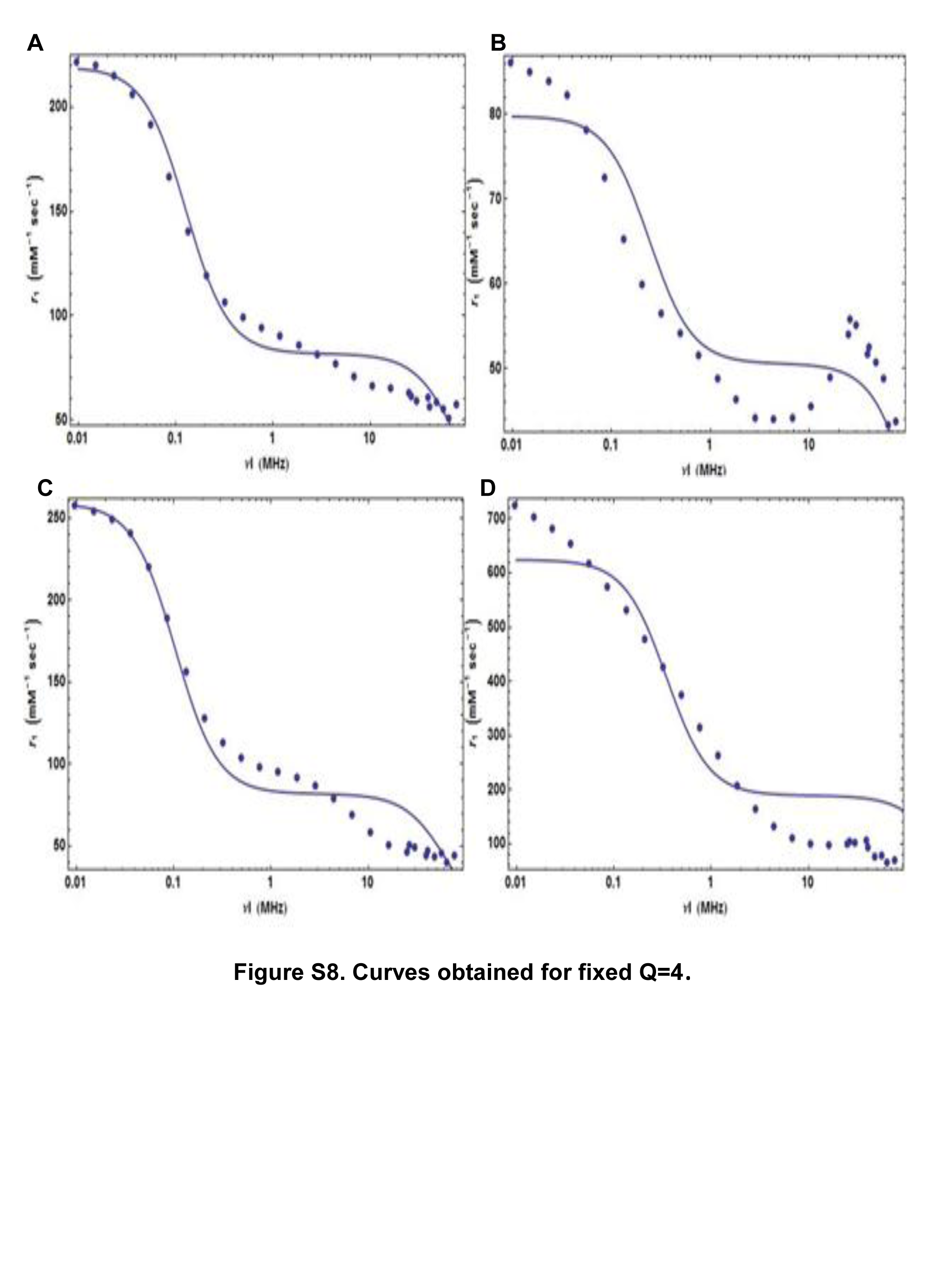

Supplement: Figure S8 — Curves obtained for fixed Q = 4 with remaining SBM parameters allowed to float. A) Oxidized Graphite, B) Oxidized Graphene Nanoplatelets, C) Reduced Graphene Nanoplatelets, D) Graphene Nanoribbons. (TIF) [file pone.0038185.s008.tif]

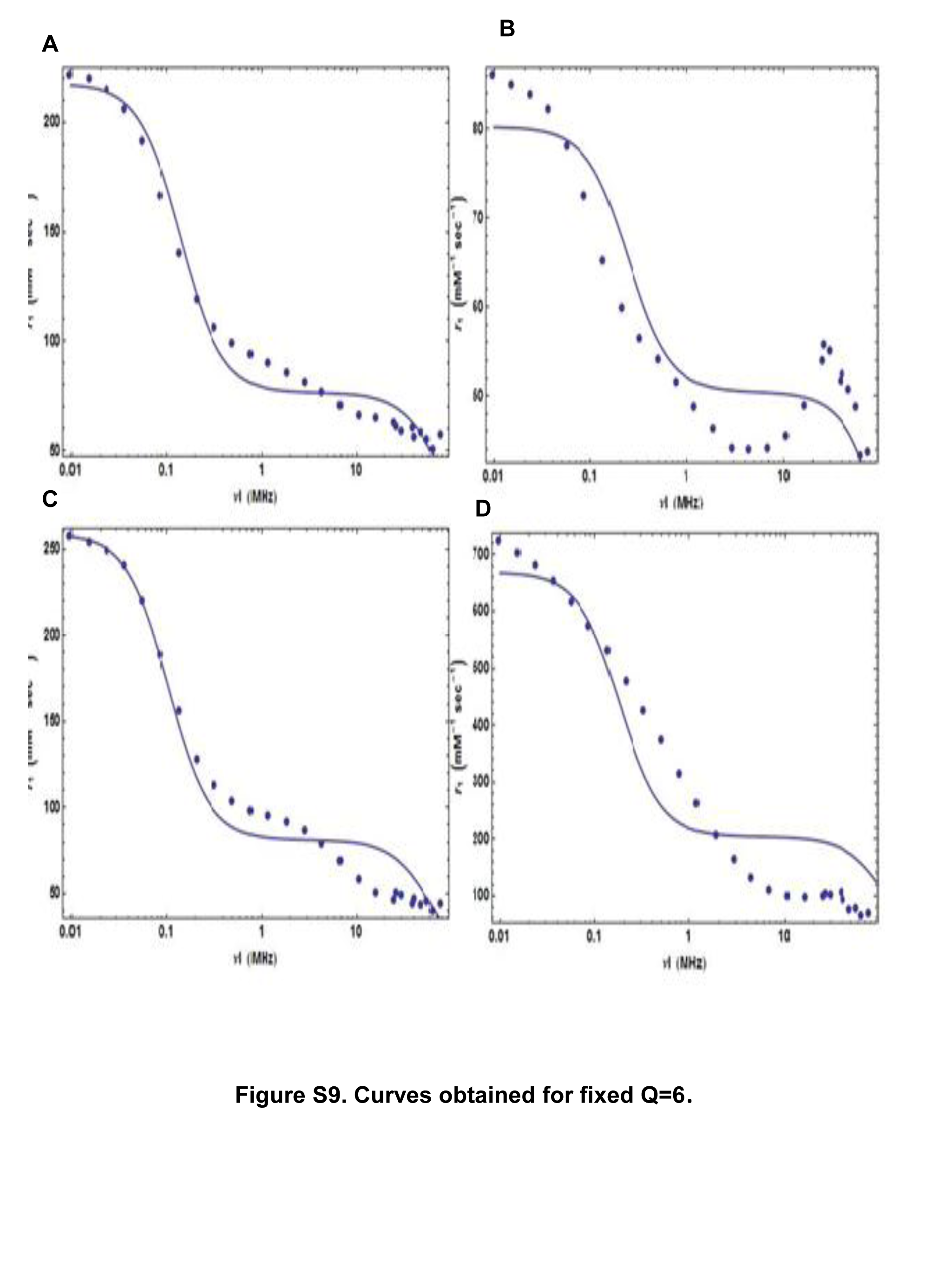

Supplement: Figure S9 — Curves obtained for fixed Q = 6 with remaining SBM parameters allowed to float. A) Oxidized Graphite, B) Oxidized Graphene Nanoplatelets, C) Reduced Graphene Nanoplatelets, D) Graphene Nanoribbons. (TIF) [file pone.0038185.s009.tif]

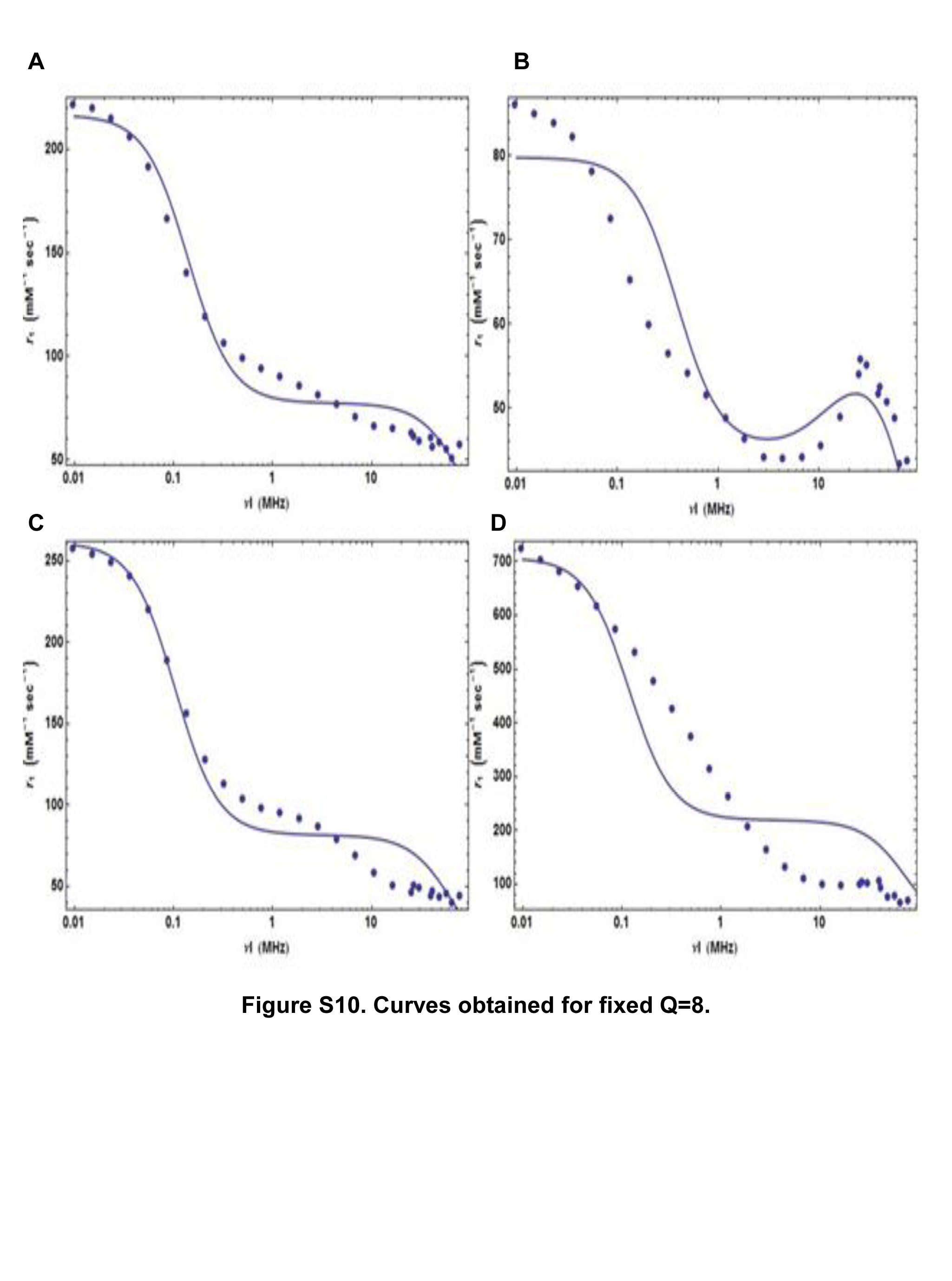

Supplement: Figure S10 — Curves obtained for fixed Q = 8 with remaining SBM parameters allowed to float. A) Oxidized Graphite, B) Oxidized Graphene Nanoplatelets, C) Reduced Graphene Nanoplatelets, D) Graphene Nanoribbons. (TIF) [file pone.0038185.s010.tif]

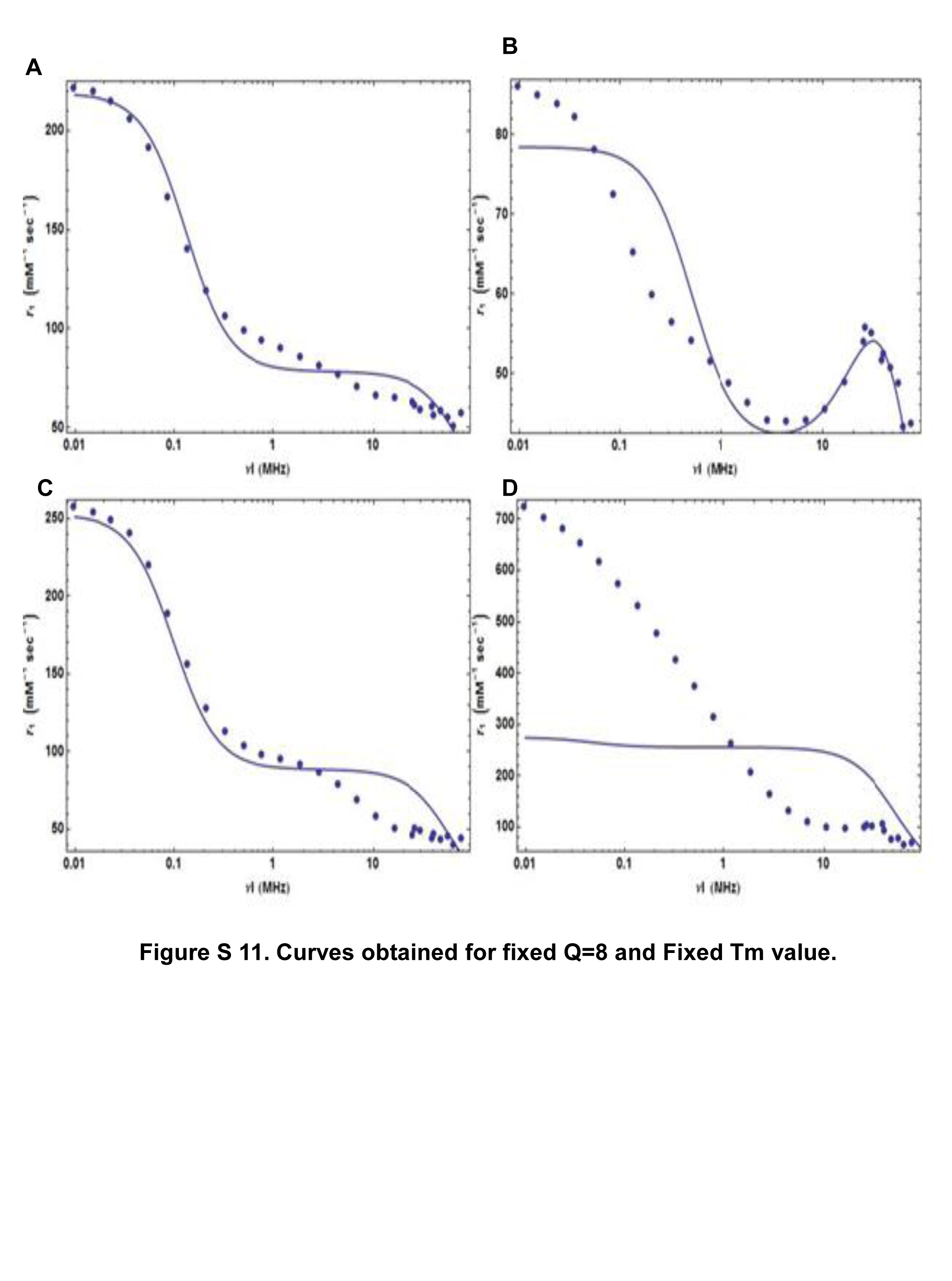

Supplement: Figure S11 — Curves obtained for fixed Q = 8 and Fixed Tm at values shown in Table S3, with remaining SBM parameters allowed to float. A) Oxidized Graphite, B) Oxidized Graphene Nanoplatelets, C) Reduced Graphene Nanoplatelets, D) Graphene Nanoribbons. The fit for the Graphene Nanoribbons in D is surprisingly worse than expected. (TIF) [file pone.0038185.s011.tif]
