# Supplementary material for: Physicochemical Characterization, and Relaxometry Studies of Micro-Graphite Oxide, Graphene Nanoplatelets, and Nanoribbons
Source: PLoS One. 2012 Jun 7;7(6):e38185. doi: 10.1371/journal.pone.0038185 (PMC3369907; doi:10.1371/journal.pone.0038185)
Supplement: Table S1 — Trace elemental analysis of solid samples of the oxidize micro-graphite, oxidized graphene nanoplatelets, reduced graphene nanoplatelets and graphene nanoribbons. The standard deviation among the various batches was 10%. (DOCX) [file pone.0038185.s012.docx]

**Table S1.** Trace elemental analysis of solid samples of the oxidize micro-graphite, oxidized graphene nanoplatelets, reduced graphene nanoplatelets and graphene nanoribbons. The standard deviation among the various batches was 10%.

| **Sample** | **Potassium (wt %)** | **Manganese (wt %)** | **Iron (wt %)** |
| --- | --- | --- | --- |
| Solid oxidized graphite | 0.52 | 3.84 | - |
| Solid oxidized graphene nanoplatelets | 0.45 | 4.54 | - |
| Solid reduced graphene nanoplatelets | 0.22 | 5.11 | - |
| Solid graphene nanoribbons | 0.29 | 0.93 | 0.005 |
