# Supplementary material for: Physicochemical Characterization, and Relaxometry Studies of Micro-Graphite Oxide, Graphene Nanoplatelets, and Nanoribbons
Source: PLoS One. 2012 Jun 7;7(6):e38185. doi: 10.1371/journal.pone.0038185 (PMC3369907; doi:10.1371/journal.pone.0038185)
Supplement: Table S2 — Trace elemental analysis of aqueous samples of the oxidized micrographite, oxidized graphene nanoplatelets, reduced graphene nanoplatelets and graphene nanoribbons. The values presented are for one batch of samples. (DOCX) [file pone.0038185.s013.docx]

**Table S2.** Trace elemental analysis of aqueous samples of the oxidized micrographite, oxidized graphene nanoplatelets, reduced graphene nanoplatelets and graphene nanoribbons. The values presented are for one batch of samples.

| **Sample** | **Manganese (ppm)** |
| --- | --- |
| Aqueous oxidized graphite | 0.82 |
| Aqueous oxidized graphene nanoplatelets | 1.48 |
| Aqueous reduced graphene nanoplatelets | 0.60 |
| Aqueous graphene nanoribbons | 0.27 |
