# Supplementary material for: Physicochemical Characterization, and Relaxometry Studies of Micro-Graphite Oxide, Graphene Nanoplatelets, and Nanoribbons
Source: PLoS One. 2012 Jun 7;7(6):e38185. doi: 10.1371/journal.pone.0038185 (PMC3369907; doi:10.1371/journal.pone.0038185)
Supplement: Table S3 — List of parameter values in SBM equations that are fixed constants, or independently established physical quantities. (DOCX) [file pone.0038185.s014.docx]

**Table S3.** List of parameter values in SBM equations that are fixed constants, or independently established physical quantities.

| **Parameter** | **Definition** | **Value** |
| --- | --- | --- |
| $\gamma_{I}$ | Gyromagnetic constant for protons | 2.675x10^8^ T^-1^S^-1^ |
| $g$ | Electronic *g* factor | 2 |
| $\mu_{B}$ | Bohr magneton | 9.274x10^-24^JT^-1^ |
| $\frac{\mu_{0}}{4\pi}$ | Free-space permeability constant | 10^-7^ NA^-2^ |
|  | Hyperfine coupling constant | 1 MHz |
|  | Reduced Planck’s constant | 1.054x10^-34^ |
| $\nu_{I}$ | Proton Larmor Frequency | $\gamma_{I}B/(2\pi)$ |
| $\nu_{S}$ | Electron Larmor Frequency | $658\times\nu_{I}$ |
| $S$ | Spin number | 5/2 |
