# Supplementary material for: Physicochemical Characterization, and Relaxometry Studies of Micro-Graphite Oxide, Graphene Nanoplatelets, and Nanoribbons
Source: PLoS One. 2012 Jun 7;7(6):e38185. doi: 10.1371/journal.pone.0038185 (PMC3369907; doi:10.1371/journal.pone.0038185)
Supplement: Table S4 — SBM Parameters obtained from the curve fit with all parameter values floating. (DOCX) [file pone.0038185.s015.docx]

**Table S4**. SBM Parameters obtained from the curve fit with all parameter values floating.

| **Parameter** | **Definition** | **Oxidized Graphite** | **Graphene Nanoplatelets** | **Reduced Graphene Nanoplatelets** | **Graphene Nanoribbons** |
| --- | --- | --- | --- | --- | --- |
| $\Delta^{2}$ | Zero-field splitting energy (ZFS) | $1.29\times10^{18}$ | $1.0\times10^{18}$ | $1.0\times10^{18}$ | $1.0\times10^{18}$ |
| $r_{MnH}$ | Manganese-Hydrogen Bond Radius | $4.03\times10^{-10}$ | $3.16\times10^{-10}$ | $3.07\times10^{-10}$ | $2.03\times10^{-10}$ |
| $q$ | Hydration number | $11.03$ | $4.58$ | $1.71$ | $1.21$ |
| $\tau_{R}$ | Tumbling time of complex | $2.16\times10^{-9}$ | $1.35\times10^{-9}$ | $4.42\times10^{-9}$ | $6.07\times10^{-9}$ |
| $\tau_{V}$ | Correlation time for splitting | $1.0\times10^{-12}$ | $2.79\times10^{-12}$ | $3.18\times10^{-12}$ | $1.00\times10^{-12}$ |
| $\tau_{M}$ | Residence time of inner sphere water molecules | $1.82\times10^{-7}$ | $7.53\times10^{-7}$ | $8.36\times10^{-9}$ | $8.09\times10^{-10}$ |
