# Supplementary material for: Physicochemical Characterization, and Relaxometry Studies of Micro-Graphite Oxide, Graphene Nanoplatelets, and Nanoribbons
Source: PLoS One. 2012 Jun 7;7(6):e38185. doi: 10.1371/journal.pone.0038185 (PMC3369907; doi:10.1371/journal.pone.0038185)
Supplement: Table S6 — SBM Parameters obtained from the curve fit for fixed Q = 4 and remaining SBM parameters allowed to float. (DOCX) [file pone.0038185.s017.docx]

**Table S6**. SBM Parameters obtained from the curve fit for fixed Q=4 and remaining SBM parameters allowed to float.

| **Parameter** | **Definition** | **Oxidized Graphite** | **Graphene Nanoplatelets** | **Reduced Graphene Nanoplatelets** | **Graphene Nanoribbons** |
| --- | --- | --- | --- | --- | --- |
| $\Delta^{2}$ | Zero-field splitting energy (ZFS) | 1.0x10^18^ | 1.0x10^18^ | 1.0x10^18^ | 1.0x10^18^ |
| $r_{MnH}$ | Manganese-Hydrogen Bond Radius | 3.37x10^-10^ | 3.13x10-^10^ | 3.51x10^-10^ | 2.47x10^-10^ |
| $q$ | Hydration number | 4 | 4 | 4 | 4 |
| $\tau_{R}$ | Tumbling time of complex | 2.21x10^-9^ | 1.48x10^-9^ | 3.57x10^-9^ | 4.83x10^-9^ |
| $\tau_{V}$ | Correlation time for splitting | 1.0x10^-12^ | 1.0x10^-12^ | 1.99x10^-12^ | 1.0x10^-12^ |
| $\tau_{M}$ | Residence time of inner sphere water molecules | 8.69x10^-8^ | 6.76x10^-7^ | 8.54x10^-9^ | 8.23x10^-10^ |
