# Supplementary material for: Physicochemical Characterization, and Relaxometry Studies of Micro-Graphite Oxide, Graphene Nanoplatelets, and Nanoribbons
Source: PLoS One. 2012 Jun 7;7(6):e38185. doi: 10.1371/journal.pone.0038185 (PMC3369907; doi:10.1371/journal.pone.0038185)
Supplement: Table S8 — SBM Parameters obtained from the curve fit for fixed Q = 8 and remaining SBM parameters allowed to float. (DOCX) [file pone.0038185.s019.docx]

**Table S8**. SBM Parameters obtained from the curve fit for fixed Q=8 and remaining SBM parameters allowed to float.

| **Parameter** | **Definition** | **Oxidized Graphite** | **Graphene Nanoplatelets** | **Reduced Graphene Nanoplatelets** | **Graphene Nanoribbons** |
| --- | --- | --- | --- | --- | --- |
| $\Delta^{2}$ | Zero-field splitting energy (ZFS) | 1.0x10^48^ | 6.12x10^18^ | 1.0x10^18^ | 1.0x10^18^ |
| $r_{MnH}$ | Manganese-Hydrogen Bond Radius | 3.76x10^-10^ | 3.73x10^-10^ | 3.94x10^-10^ | 3.26x10^-10^ |
| $q$ | Hydration number | 8 | 8 | 8 | 8 |
| $\tau_{R}$ | Tumbling time of complex | 1.95x10^-9^ | 1.77x10^-9^ | 3.85x10^-9^ | 3.69x10^-9^ |
| $\tau_{V}$ | Correlation time for splitting | 1.18x10^-12^ | 1.09x10^-11^ | 1.99x10^-12^ | 1.0x10^-12^ |
| $\tau_{M}$ | Residence time of inner sphere water molecules | 1.42x10^-7^ | 7.29x10^-7^ | 7.06x10^-9^ | 5.06x10^-9^ |
