# Supplementary material for: Physicochemical Characterization, and Relaxometry Studies of Micro-Graphite Oxide, Graphene Nanoplatelets, and Nanoribbons
Source: PLoS One. 2012 Jun 7;7(6):e38185. doi: 10.1371/journal.pone.0038185 (PMC3369907; doi:10.1371/journal.pone.0038185)
Supplement: Table S9 — SBM Parameters used to obtain curve fit for fixed Q = 8 and fixed Tm values. (DOCX) [file pone.0038185.s020.docx]

**Table S9**. SBM Parameters used to obtain curve fit for fixed Q=8 and fixed Tm values.

| **Parameter** | **Definition** | **Oxidized Graphite** | **Graphene Nanoplatelets** | **Reduced Graphene Nanoplatelets** | **Graphene Nanoribbons** |
| --- | --- | --- | --- | --- | --- |
| $\Delta^{2}$ | Zero-field splitting energy (ZFS) | 1.0x10^18^ | 1.80x10^19^ | 1.0x10^18^ | 1.0x10^18^ |
| $r_{MnH}$ | Manganese-Hydrogen Bond Radius | 3.79x10^-10^ | 3.87x10^-10^ | 3.90x10^-10^ | 2.79x10^-10^ |
| $q$ | Hydration number | 8 | 8 | 8 | 8 |
| $\tau_{R}$ | Tumbling time of complex | 2.07x10^-9^ | 2.21x10^-9^ | 2.79x10^-9^ | 1.0x10^-8^ |
| $\tau_{V}$ | Correlation time for splitting | 1.0x10^-12^ | 6.93x10^-12^ | 1.0x10^-12^ | 1.0x10^-12^ |
| $\tau_{M}$ | Residence time of inner sphere water molecules | 1.42x10^-7^ | 1.29x10^-7^ | 1.06x10^-7^ | 5.06x10^-7^ |
