# Supplementary material for: Physicochemical Characterization, and Relaxometry Studies of Micro-Graphite Oxide, Graphene Nanoplatelets, and Nanoribbons
Source: PLoS One. 2012 Jun 7;7(6):e38185. doi: 10.1371/journal.pone.0038185 (PMC3369907; doi:10.1371/journal.pone.0038185)
Supplement: Text S1 — Information on structural characterization, elemental and Raman analysis, Solomon-Bloembergan-Morgan Theory of Relaxivity and 17O-transverse relaxation rate measurements on oxidized micro graphite, graphene nanoplatelet and nanoribbons. (DOCX) [file pone.0038185.s021.docx]

**Supplementary Information for**

**Physicochemical characterization and relaxometry studies of micro-graphite oxide, graphene nanoplatelets and nanoribbons**

Bhavna S Paratala,^1^ Barry D Jacobson,^1^ Shruti Kanakia,^1^ Francis Leonard Deepak,^2^ Balaji Sitharaman^1*^

^1^Department of Biomedical Engineering,

Stony Brook University,

Stony Brook, NY 11794-5281, USA

^*^Email: [bsitharaman@notes.cc.sunsyb.edu](mailto:bsitharaman@notes.cc.sunsyb.edu)

^2^International Iberian Nanotechnology Laboratory,

Avda Mestre Jose Veiga, Braga 4715, Portugal

**1. Structural Characterization and Raman Analysis**

**Method**

Scanning electron microscopy (SEM, JSM 5300, JEOL) was performed at 80 kV on the oxidized micro-graphite samples to characterize their size and structure. High Resolution Transmission Electron Microscopy (TEM) imaging analysis was performed on the graphene nanoplatelets and nanoribbons samples using a high resolution analytical transmission electron microscope (JEOL JEM2010F (FEG-TEM)). Imaging was carried out at 200kV accelerating voltage. TEM samples were prepared by dispersing the dry powders in 1:1 ethanol:water to form a homogeneous mixture. The suspension was then deposited on to a 300 mesh Cu grid covered with a lacey carbon film. For the aberration (C_s_) corrected TEM characterization, the experiments were performed in a Titan cubed 300-60 kV operated at 80 kV equipped with a spherical aberration corrector for the objective lens. Images were commonly recorded for 0.4 seconds. The Electronic Energy Loss Spectra (EELS) detector in this case used to collect the spectra was Tridiem. RAMAN spectral analysis of graphite, oxidized graphite, and all graphene samples was performed between 200 to 3000cm^-1^ using a Thermo Scientific DXR Raman confocal microscope at 530 nm diode laser excitation wavelength and room temperature.

**Results and Discussion**

Figure S1a displays the scanning electron microscopy image of the oxidized micro-graphite particles. The images indicate that oxidized micrographite particles exist as fractured structure, and have sizes (longest length of fractured structure) between 3-4 µm with an average size of 2.5 µm. Figures S1b and S1c display representative low and high magnification TEM images of reduced graphene nanoplatelets, respectively, which provide their structural and morphological information. The structural properties of the graphene nanoparticles are similar to recent reports on the large scale production of graphene nanoplatelets and graphene nanoribbons [[1-7](#_ENREF_1)]. As seen in Figure S1b, the reduced graphene nanoplatelets are circular in shape with an average width of ~20 nm. Some platelets appear darker than the others, and this is due to the presence of multi-layered graphene sheets. The lighter ones, which are almost transparent, are single or double layered graphene sheets. Figure S1c reveals the atomic lattice fringe structure of the individual graphene sheets; the lattice grid lines and hexagonal carbon atom rings are clearly visible [[8](#_ENREF_8)]. AFM section analysis of the reduced graphene nanoplatelets dispersion on a Si substrate revealed a uniform thickness of ~1.137nm (Figure S2c). Pristine graphene sheets have an atomic layer thickness (Van der Waals) of 0.34 nm. The presence of covalent bonds with carboxyl and hydroxyl groups, and displacement of sp^3^ carbon atoms in the graphene nanoplatelet structure has been reported to be the reason for the increase in the thickness [[2](#_ENREF_2)]. Oxidized graphene oxide nanoparticles show similar sizes and architecture (Figure S2b).

Figure S1d and S2a display representative low and high magnification TEM images of graphene nanoribbons, respectively. As seen in Figure S1d, the graphene nanoribbons have fully unzipped layers of graphene sheets. The high resolution TEM image in Figure S2a clearly shows that the nanoribbons are multilayered (arrows) due to successive unzipping of the concentric walls of MWCNTs. The graphene oxide nanoribbons structure appears mostly uniform and smooth, with few defects. The starting material, MWCNTs, have an outer diameter of 40- 70 nm, and length of 500 -2000 nm. Since the MWCNTs are cylinders, upon unzipping, they should open up completely to have breadths of ~ 125 – 220 nm (π x diameter) and lengths of 500- 2000 nm. The analysis of the TEM images indicates that the width of the graphene nanoribbons is ~120 nm which is greater than the outer diameter of the outermost tubes of MWCNTs of 70 nm verifying the process of unzipping. However, this width is slightly lower than the range required for fully flat ribbons (125- 220 nm) suggesting that, the graphene nanoribbons upon unzipping may not be fully flat sheets, but retain some curvature of the MWCNTs. The TEM images also show that the graphene nanoribbons have lengths of ~600- 2000 nm similar to the MWCNTs.

Figure S3a shows the Raman spectra of oxidized micro-graphite, oxidized graphene nanoplatelets and reduced graphene nanoplatelets. Also included as control, is the Raman spectra of pristine micro-graphite. The spectrum of pristine micro-graphite shows a prominent sharp peak at 1581cm^-1^ indicating the G-band which is attributed to the doubly degenerate zone center E_2g_ mode[[9](#_ENREF_9)]. In case of oxidized graphite, there is a broadening of the G band, and a peak shift to 1595cm^-1^. Further, zone boundary phonons give rise to the D band at 1345cm^-1^, which becomes prominent indicating increase in the disorder sp^2^ domains, and reduction of the crystal size due to oxidation. Due to oxidation of graphite, there is an increase in the ratio of intensity of the D to G peaks (I_D_/ I_G_), from 0.407 for graphite to 1.2 for oxidized graphite [[9](#_ENREF_9)]. The spectra of oxidized graphene nanoplatelets, and reduced graphene nanoplatelets show a further increase in I_D_/ I_G_ to 1.3 and 1.44, respectively. In case of reduced graphene nanoplatelets, the peaks of D and G bands are shifted closer to the values of graphite (1330cm^-1^ and 1590cm^-1^ respectively), suggesting the removal of the oxygen during reduction, and some restoration of sp^2^ carbon atoms. However, I_D_/ I_G_ ratio is higher compared to oxidized graphene nanoplatelets possibly due to the reduction of the average size of sp^2^ domains in addition to an increase in the number of such small sized disorder domains [[2](#_ENREF_2)].

Figure S3b shows the Raman spectrum of graphene nanoribbons and MWCNTs. The spectrum for graphene nanoribbons has a broad G band, which is red-shifted at 1600 cm^-1^ compared to MWCNT and has a prominent D band at 1310 cm^-1^. There is an increase in I_D_/ I_G_ value from 0.045 for MWCNTs to 1.57 for the graphene nanoribbons, similar to previous reports [[5](#_ENREF_5)]. The red-shift in the G band for the graphene nanoribbons is due to the oxidative unzipping of MWCNTs, and is similar to the shift in spectra for oxidized graphene nanoplatelets, due to oxidation of graphite (Figure S3a).

The Raman spectra of reduced graphene nanoplatelets also show additional peaks at around 657cm^-1^, 370cm^-1^ and 320cm^-1^ (Figure S3c). In order to identify the peaks, a Raman spectral database search (using the RRUFF™ project collection, http://rruff.info/R040090) attributed the peaks to Hausmannite (Mn_3_O_4_); a complex oxide containing di-valent and tri-valent manganese. Hausmannite is the most stable oxide of manganese, and is formed when any other oxides, hydroxides, carbonates, nitrates or sulphates of manganese are calcinated [[10-12](#_ENREF_10)]. In our case, the high temperature (~100 °C) heating during the synthesis of the reduced graphene nanoplatelets may have led to hausmannite formation. The detection of hausmannite peaks was sensitive to the orientation of the sample, and sample spot size indicating of its presence in very small amounts. The EPR spectra (see main manuscript Figure 3 and 4) of the sample also did not detect any Mn (III) ion, further corroborating that hausmannite may be present in relatively small amounts compared to oxides of divalent manganese.

Unlike reduced graphene nanoplatelets, no hausmannite peaks were detected in Raman spectra of the oxidized micro-graphite, oxidized graphene nanoplatelets or nanoribbons samples. Electron energy loss spectroscopy (EELS) of oxidized and reduced graphene nanoplatelets, (Figure S4) detected manganese and oxygen. However, EELS spectroscopy of graphene nanoribbons (at the center or the edges) did not show any manganese. Additionally, trace elemental analysis (Table S1,S2) of all the samples (oxidized micrographite, oxidized graphene nanoplatelets, reduced graphene nanoplatelets and graphene nanoribbons) detected the presence of manganese. Thus, the Raman spectroscopy results taken together with EELS and elemental analysis measurements indicate that, in case of oxidized micro-graphite, oxidized graphene nanoplatelets, reduced graphene nanoplatelets and graphene nanoribbons, divalent manganese in the form of manganese sulfate or manganese oxide maybe intercalated between graphene layers, since the reaction of potassium permanganate with sulfuric acid leads to formation of divalent manganese. Additionally, trace amounts of hausmannite may be intercalated between the graphene layers for reduced graphene nanoplatelets [[13](#_ENREF_13)].

**2. Elemental Analysis**

**Methods**

The solid and liquid graphene nanoplatelets and nanoribbon samples were analyzed by Inductively-coupled plasma optical emission spectroscopy (ICP-OES) at two micro-analytical analytical testing laboratories (Columbia Analytical Services, Tucson, AZ and Galbraith Laboratories, Inc., Knoxville, TN) to confirm, and determine the concentration of manganese and potassium. Additionally, iron content analysis was carried out for the graphene nanoribbon samples, since iron is used as a catalyst in the preparation of MWCNTs (the starting material). For the ICP analysis, solid and liquid graphene nanoplatelets and nanoribbon samples (known weight or concentration) were treated with concentrated HNO_3_, and carefully heated to obtain a solid residue. They were next treated with 30%H_2_O_2_, and heated again to remove any carbonaceous material. The remaining solid residue was dissolved in 2%HNO_3_, and analyzed by ICP.

**Results and Discussion**

Table S1 and S2 presents the trace elemental analysis of solid and aqueous samples, respectively, of the oxidized micro-graphite, oxidized graphene nanoplatelets, reduced graphene nanoplatelets and graphene nanoribbons. For the solid samples (Table S1), since potassium permanganate was used in the preparation of these nanoparticles, the concentration of potassium and manganese in these samples were analyzed. Additionally, iron elemental analysis was also performed on the graphene nanoribbons, since iron catalysts were present in the MWCNTs; the starting material used in the graphene nanoribbon preparation. All the solid samples showed potassium between 0.22-0.52 wt%. Graphene nanoribbons showed at least 4 times lower amounts of manganese (0.93 wt%) compared to the other solid samples which showed manganese between 3.84-5.11 wt%. For the aqueous samples (Table S2), concentrations of manganese were analyzed for all samples, since they are needed for the calculation of the relaxivity of these samples. For the graphene nanoribbons solutions, iron elemental analysis was also performed as it could also contribute to the calculated relaxivity values. The concentrations of manganese in the all aqueous samples were variable between 0.27-1.48 ppm. This broad range of values in concentration is due to the variable propensity of the different samples (see method section on proton relaxivity measurement) to form stable suspensions in 1% Pluronic F127 solution. No iron was detected in the aqueous solutions of graphene nanoribbons. This non-detection of iron may be due to the following reason. The concentration of the graphene nanoribbons used for the relaxivity is 10μg/ml. A 300μl volume solution was used for the relaxivity experiments, and the trace elemental analysis. Thus, the total amount of graphene nanoribbons is 3μg. If one considers Fe concentration to 0.005% of 3μg, the amount of Fe would be 0.15 ng, which is well below the detection limit of ICP system (detection limit ~ 1 ng).

**3.**

Following are the set of SBM equations.[[14](#_ENREF_14)]

where $P_{m}$, the mole fraction of Mn, is defined in Equation 13.

$T_{1m}$ and $T_{2m}$, the net proton relaxation times, are given by:

$C_{dd}$ contains the physical constants which govern dipole-dipole interactions: $\tau_{c1}$

$$C_{DD}=\frac{2}{15}\frac{\gamma_{I}^{2}g^{2}\mu_{B}^{2}}{r_{Gd-H}^{6}}\left(\frac{\mu_{0}}{4\pi}\right)^{2}S\left(S+1\right)$$

The relaxation effectiveness of the paramagnetic centers are:

As before, the transfer efficiency from the paramagnetic center to the H protons via dipole-dipole interactions is mediated by the correlation times $\tau_{c1}$and $\tau_{c2}$ which are given by:

For scalar interactions the relevant correlation time is $\tau_{e}$ which is given by:

$$\frac{1}{\tau_{e}}=\frac{1}{T_{2e}}+\frac{1}{\tau_{m}}$$

Finally, we have

$$\omega_{I}=2\pi\nu_{I}$$

and

$$\omega_{S}=658\omega_{I}=658\left(2\pi\nu_{I}\right)$$

where $\omega_{I}$ and $\omega_{S}$ are the Larmor frequencies of the paramagnetic metal’s electron spin, and the water proton’s nuclear spin, respectively.

$P_{M}$is the mole fraction of the Manganese (Mn^+2^) with respect to the total number of moles of Mn^+2^ and water (H_2_O). The concentration of Mn^+2^ used here was 1 mM.

$$P_{M}=\frac{m_{Mn}}{m_{Mn}+m_{H_{2}O}}\approx\frac{m_{Mn}}{m_{H_{2}O}}=\frac{10^{-3}}{55.56}=1.8\times10^{-5}$$

The remaining physical constants in the above equations are given below in Table S3.

The mechanism by which paramagnetic complexes improve relaxivity is via coupling of the electron spin of the paramagnetic ion to the proton spin. This coupling occurs by two primary methods: scalar (through bonds) and dipole-dipole (DD) (through space) interactions. DD interactions are generally stronger, but depend on the orientation of the spin system of the paramagnetic ion with respect to the orientation of the H atoms in the water molecule. Since the molecules are continually tumbling with respect to each other, the rotational coherence time $\tau_{R}$ which is roughly a measure of the time the molecules rotate by a radian with respect to each other, is an important factor for DD interactions. The longer the$\tau_{R}$, the more effective is the influence of the paramagnetic center. However, for scalar coupling, the physical orientation is irrelevant, as the influence is exerted through the bonds of the compound. For this reason, $\tau_{R}$ is present in Equations 8 and 9, above, which govern DD interactions for $T_{1}$ and$T_{2}$, respectively, but is absent in Equation 10, which governs scalar interactions. The total strength of interaction is the sum of the DD and scalar contributions, and is reflected in Equation 3, where the first term represents the DD contribution, and the second term, the scalar contribution.

A key factor in modeling the contribution of the inner sphere is to identify the number of water molecules that can bind to the paramagnetic center at any given time. Equation 1 tells us that the relaxivity is directly proportional to this hydration number, $q$. Another point which is apparent from Equation 1 is that aside from the concentration of the contrast agent, the relaxivities $r_{1}$ and $r_{2}$ are determined by the total relaxation times of the bound inner sphere water molecules $T_{1m}$ and $T_{2m}$ respectively, and by the residence lifetime $\tau_{m}$, the length of time the water molecule stays bound to the paramagnetic center before detachment and replacement by another water molecule. In turn, the factors $T_{1m}$ and $T_{2m}$ are dependent on the factors $T_{1e}$ and$T_{2e}$, which are the electron relaxation times of the paramagnetic center. These are defined in Equations 6 and 7 respectively, for the longitudinal and transverse cases, and depend among other things upon the applied field. The effectiveness of the transfer of relaxivity from the electrons of the paramagnetic center to the protons is governed by Equations 8 and 9 for the DD case, and Equation 10 for the scalar case. Aside from the strength of the paramagnetic agent, Equations 8 and 9 tell us that the effectiveness of transfer of the RF fields generated by the electrons of the contrast agent to the protons is also a critical factor in the overall relaxivity. This transfer is mediated by the tumbling time $\tau_{R}$ and the residence lifetime$\tau_{m}$. The longer these are, the more effective the transfer.

The SBM equations were fit to the experimental data using the least squares algorithm (FindFit in Mathematica®*)*. Constraints were used to limit the possible solutions, as curve-fitting algorithms are notorious for producing physically unrealizable or meaningless solutions. The data was also fit to the Levenberg-Marquardt algorithm which produced better fits, but the returned parameters were often nonsensical, such as negative values, and/or differing by many orders of magnitude from accepted values. Because the Levenberg-Marquardt algorithm cannot be used with preset constraints, the minimize option in the FindFit function was used that allow the use of constraints, and returned results rapidly. It should also be noted that while fitting the NMRD data, the parameters returned by the algorithm may represent only a local minimum, and not the global minimum. It is possible that better solutions may exist. However, these are very difficult to locate and verify. In addition, slight adjustments to one parameter can cause widely fluctuating changes in the other parameters.

**Results and Discussion**

We performed a number of curve fitting experiments to best analyze the NMRD data for each of the four materials reported here. There is a tradeoff between the number of variables that are allowed to float, and are computed by the curve-fitting algorithm, and the numbers that are assumed fixed, and which have been determined by other means. Independent corroboration of some variables generally produces more accurate values for those parameters, but may adversely affect the tightness of fit. Conversely, allowing the algorithm to find all parameters often leads to an excellent fit, but occasionally to physically meaningless results, including negative values of time. To limit these occurrences, we generally constrained the desired parameters to lie within physically reasonable ranges during the running of the algorithm.

To corroborate some of the SBM parameters, we independently determined values for q and $\tau_{M}$by EPR and ^17^O-transverse relaxation rate measurements. The value of q that was obtained was 8 for all samples, and the values of $\tau_{M}$were oxidized graphite = 200 ns, oxidized graphene nanoplatelets = 500 ns, reduced graphene nanoplatelets = 350 ns and graphene nanoribbons = 400 ns.

The best fit was obtained for q = 8 which is corroborated by the EPR measurements. However, we have considered the possibility where Mn(II) ions are co-ordinated to graphene sheet or oxygen atoms and also obtained fits for q = 2, 4 and 6 as well as floated the values of q.

The following fitting strategies were employed.

1. Float all SBM parameters (Figure S6).
2. Fix Q at 2, Float remaining SBM parameters (Figure S7).
3. Fix Q at 4, Float remaining SBM parameters (Figure S8).
4. Fix Q at 6, Float remaining SBM parameters (Figure S9).
5. Fix Q at 8, Float remaining SBM parameters (Figure S10).
6. Fix Q at 8, Fix Tm, Float remaining SBM parameters (Figure S11).

**4.^17^O-transverse relaxation rate measurements**

A Bruker Avance 500 spectrometer was used for the ^17^O measurements. Experimental settings were: no sample spinning, spectral width 10 kHz, 90°pulse, acquisition time 25 ms, and 256 scans. CD_3_CN contained in a capillary coaxially inserted in the 5 mm tube containing the experimental sample was as used to carry out the field-frequency lock. The experimental solutions were enriched in ^17^O isotope (to 3 %) by adding 17O enriched water (10 % H_2_^17^O) to improve the detection sensitivity. The linewidth at half height of the water ^17^O signal was measured, and this value was used to calculate ^17^O-transverse relaxation rate measuring (*R_2_* = π x linewidth at half height). The water exchange correlation time ($\tau_{M}$) was estimated from the analysis of the temperature dependence (between 15-80 °C) of the transverse relaxation rate for the four samples dispersed in ^17^O-water using the Swift and Connick theory [[15](#_ENREF_15)]. At 27 °C, the $\tau_{M}$values for the four samples were as follows. Oxidized graphite = 200 ns, oxidized graphene nanoplatelets = 500 ns, reduced graphene nanoplatelets = 350 ns and graphene nanoribbons = 400 ns.

**References**

1. Stankovich S, Piner R, Chen X, Wu N, Nguyen S, et al. (2006) Stable aqueous dispersions of graphitic nanoplatelets via the reduction of exfoliated graphite oxide in the presence of poly (sodium 4-styrenesulfonate). Journal of Materials Chemistry 16: 155-158.

2. Stankovich S, Dikin D, Piner R, Kohlhaas K, Kleinhammes A, et al. (2007) Synthesis of graphene-based nanosheets via chemical reduction of exfoliated graphite oxide. Carbon 45: 1558-1565.

3. Stankovich S, Piner R, Nguyen S, Ruoff R (2006) Synthesis and exfoliation of isocyanate-treated graphene oxide nanoplatelets. Carbon 44: 3342-3347.

4. Li D, Müller M, Gilje S, Kaner R, Wallace G (2008) Processable aqueous dispersions of graphene nanosheets. Nature nanotechnology 3: 101-105.

5. Kosynkin D, Higginbotham A, Sinitskii A, Lomeda J, Dimiev A, et al. (2009) Longitudinal unzipping of carbon nanotubes to form graphene nanoribbons. Nature 458: 872-876.

6. Higginbotham A, Kosynkin D, Sinitskii A, Sun Z, Tour J (2010) Lower-defect graphene oxide nanoribbons from multiwalled carbon nanotubes. ACS nano 4: 2059-2069.

7. Geng Y, Wang S, Kim J (2009) Preparation of graphite nanoplatelets and graphene sheets. Journal of colloid and interface science 336: 592-598.

8. Lu G, Mao S, Park S, Ruoff R, Chen J (2009) Facile, noncovalent decoration of graphene oxide sheets with nanocrystals. Nano Research 2: 192-200.

9. Tuinstra F, Koenig J (1970) Raman spectrum of graphite. The Journal of Chemical Physics 53: 1126.

10. Southard J, Moore G (1942) High-temperature Heat Content of Mn3O4, MnSiO3 and Mn3C1. Journal of the American Chemical Society 64: 1769-1770.

11. Ursu I, Alexandrescu R, Mihailescu I, Morjan I, Jianu V, et al. (1986) Kinetic evolution during the laser/thermal preparation of Mn3O4 from MnCO3. Journal of Physics B: Atomic and Molecular Physics 19: L825.

12. Bie X, Wang C, Ehrenberg H, Wei Y, Chen G, et al. (2010) Room-temperature ferromagnetism in pure ZnO nanoflowers. Solid State Sciences 12: 1364-1367.

13. Sorokina NE, Khaskov MA, Avdeev VV, Nikol’skaya IV (2005) Reaction of Graphite with Sulfuric Acid in the Presence of KMnO4. Russian Journal of General Chemistry 75: 162..168.

14. Toth E, Helm L, Merbach A (2001) The Chemistry of Contrast Agents in In: Merbach A, Toth E, editors. The Chemistry of Contrast Agents in Medical Magnetic Resonance Imaging: Wiley.

15. Swift TJ, Connick RE (1962) NMR-relaxation mechanisms of O^17^ in aqueous solutions of paramagnetic cations and the lifetime of water molecules in the first coordination sphere. J Chem Phys 37: 307.
